# Legume cover under *Camellia oleifera* forests enhances understory biomass carbon storage and soil CO_2_ flux but declines soil inorganic carbon storage on a karst steep slope

**DOI:** 10.3389/fmicb.2025.1714945

**Published:** 2026-01-26

**Authors:** Leilei Ding, Hang Sun, Zhongfu Long, Birong Gao, Zhenduan Zhou, Yue Ye, Song Yang, Xia Lei

**Affiliations:** 1 Guizhou Institution of Prataculture, Guizhou Academy of Agricultural Sciences, Guiyang, Guizhou, China; 2 Qiannan Comprehensive Experimental Station of China's Forage Industry Technology System, Guiyang, Guizhou, China; 3 Guizhou Key Laboratory of Agricultural Microbiology, Guizhou Academy of Agricultural Sciences, Guiyang, Guizhou, China; 4 Institute of Subtropical Crops, Guizhou Academy of Agricultural Sciences, Guiyang, Guizhou, China; 5 Guizhou Education University, Guiyang, Guizhou, China; 6 Guizhou Vocational College of Agriculture, Qingzhen, Guizhou, China

**Keywords:** community assembly, greenhouse gas, life-history strategy, microbial network, plant carbon, root carbon, soil inorganic carbon

## Abstract

**Introduction:**

Legume cover is a widely used and useful soil management strategy in orchards across the world. However, it remains unclear how the alfalfa cover affects vegetation and soil carbon storage, soil greenhouse gas fluxes, and global warming potential in *Camellia oleifera* forests. Furthermore, the understory vegetation, soil physicochemical properties, and microorganisms as potential drivers of vegetation and soil carbon storage, soil greenhouse gas fluxes, and global warming potential remain unexplored.

**Methods:**

This study determined vegetation and soil carbon storage, soil greenhouse gas fluxes, and global warming potential under legume cover and non-cropping cover and explored the potential drivers on a karst steep slope.

**Results:**

The results showed that cropping cover under *Camellia oleifera* forests elevated understory vegetation aboveground and vegetation root biomass carbon storage (*p* = 0.0002) and reduced soil N_2_O flux (*p* = 0.0210), but reduced soil inorganic carbon storage (*p* = 0.0003) and enhanced soil CO_2_ flux (*p* = 0.0002) and global warming potential (*p* = 0.0002). In addition, cropping cover not only increased understory vegetation species richness (*p* = 0.0104), aboveground biomass (*p* = 0.0002), and vegetation root biomass (*p* = 0.0009) but also decreased soil microbial carbon (*p* = 0.0360) and phosphorus limitation (*p* = 0.0104) and enhanced soil organic carbon decomposition (*p* = 0.0043). Moreover, cropping cover shifted microbial community assembly processes and life-history strategies and enhanced soil bacterial community stability (*p* = 0.0000) and soil bacterial and fungal network stability. A trade-off existed between understory vegetation aboveground (Spearman r = −0.69, *p* = 0.0030) and vegetation root biomass carbon storage (Spearman r = −0.62, *p* = 0.0099) and soil inorganic carbon storage.

**Discussion:**

Alfalfa cover is recommended for improved understory vegetation aboveground and vegetation root biomass carbon storage, soil physicochemical properties, and soil microbial community stability and network stability, whereas it may not be recommended due to increased soil CO₂ flux and global warming potential, as well as reduced soil inorganic carbon storage. This study first demonstrated the trade-off between understory vegetation aboveground and vegetation root biomass carbon storage and soil inorganic carbon storage, and this trade-off should be carefully considered when conducting multi-storage management.

## 1 Introduction

Globally, orchard area and fruit yield have increased by approximately 22 and 54%, respectively, from 2000 to 2019 (Zhao et al., 2022; Xiang et al., 2023), with orchard fruit production accounting for nearly one-third of global crop output (Hu et al., 2022). However, due to poor orchard management (Xiang et al., 2023), orchard systems release more greenhouse gasses than cereal systems (Zhao C. et al., 2021). This exacerbates global warming and hinders the pace of reducing global greenhouse gas emissions. Cropping cover in orchards has increasingly been recognized as an essential (Yao et al., 2005; Zhao C. et al., 2021; Xiang et al., 2023), environmentally friendly (Ren et al., 2023; Xiang et al., 2024), and sustainable management practice worldwide (Li et al., 2022; Ma et al., 2023). Although the effect of cropping cover has been well studied in pecan (Shi H. et al., 2024), apple (Wan and He, 2021), citrus (Tu et al., 2021), tangerine (Xiao et al., 2022), and pear (Wang et al., 2023) orchards, the effect of cropping cover in *Camellia oleifera* orchards remains poorly understood.

From the perspective of orchard vegetation, many studies have focused on the effects of cropping cover on overstory vegetation, such as water-use efficiency (Suo et al., 2019), growth (Fei et al., 2025), fruit yield (Yao et al., 2005; Suo et al., 2019; Xiang et al., 2022; Ye et al., 2022; Wang et al., 2023), and fruit quality (Ye et al., 2021; Ma et al., 2023; Ren et al., 2023; Fei et al., 2025) of trees. Limited studies on understory vegetation focus on the impact of cropping cover on groundcover (Wei et al., 2018), diversity, weed control (Li H. et al., 2023; Shi X. et al., 2024; Pedraza and Gonzalez-Andujar, 2025), and yield and feed value (Krach et al., 2025). The understory constitutes a significant portion of overall vegetation diversity (Deng et al., 2023) and has non-negligible carbon storage (Zhao Y. et al., 2021; Haq et al., 2024). However, due to limited research on understory vegetation carbon storage (Haq et al., 2024), we still know very little about the impact of cropping cover on understory vegetation carbon storage in *Camellia oleifera* forests. This may hinder the global application of cropping cover. We hypothesize that cropping cover under *Camellia oleifera* forests increases understory vegetation biomass compared to bare conditions, ultimately elevating understory vegetation carbon storage.

In addition to vegetation, an increasing number of studies suggest that cropping cover improves soil physical, chemical (Yao et al., 2005; Xiao et al., 2022; Ma et al., 2023), and biological properties (Wei et al., 2018). For instance, cover reduces soil temperature and bulk density, whereas it increases soil water content (Tang et al., 2022), organic carbon (Wei et al., 2018; Wan and He, 2021; Hu et al., 2022; Xiang et al., 2022; Xiang et al., 2023; Xiang et al., 2024), available nitrogen (Hoagland et al., 2008), phosphorus content (Wang et al., 2021; Li et al., 2022; Tang et al., 2022; Wang et al., 2023; Dong et al., 2024; Wang et al., 2024; Chen et al., 2025), and enzyme activities (Wan and He, 2021; Tang et al., 2022; Xiang et al., 2023). In addition, cropping cover enhances water and soil conservation (Fei et al., 2025) and reduces runoff (Zheng et al., 2021; Li L. et al., 2023), erosion (Duan et al., 2020; Tu et al., 2021), and nutrient loss (Tian et al., 2023). We thus hypothesize that cropping cover under *Camellia oleifera* forests improves soil physical properties and nutrient availability. As cropping cover improves soil environments (Wan and He, 2021; Xiang et al., 2024) with greater resource availability supporting microbiota (Rodriguez-Ramos et al., 2022), we further hypothesize that cropping cover under *Camellia oleifera* forests enhances soil microbiota and consequently affects soil greenhouse gas emissions and soil carbon storage. Although the impacts of different orchard management practices on soil organic carbon storage (Xiang et al., 2022) have been well studied, we still lack an understanding of the effects of cropping cover on soil inorganic carbon storage and greenhouse gas emissions in *Camellia oleifera* forests. Some studies suggest that cropping cover does not change soil greenhouse gas emissions (Chen et al., 2025), whereas others have found that tree–grass mixtures exhibit higher carbon dioxide emissions than monoculture trees (Ansari et al., 2023). Moreover, some studies suggest that cropping cover does not change soil microbial diversity (Wang R. et al., 2020; Xiao et al., 2022), whereas other studies show that cropping cover alters soil microbial community composition (Wan and He, 2021), increases soil microbial diversity (Xiao et al., 2022; Xiang et al., 2023), biomass (Dong et al., 2024), and biomass carbon (Xiang et al., 2023), and even shifts microbial life-history strategies (Wan and He, 2021) and co-occurrence networks (Xiao et al., 2022). However, there are still no studies reporting the effects of cropping cover on soil inorganic carbon storage, greenhouse gas emissions, global warming potential, and soil microbiota in *Camellia oleifera* forests. This may limit the global promotion of cropping cover under carbon reduction demands.

To fill these knowledge gaps, this study measured vegetation and soil organic and inorganic carbon storage, soil greenhouse gas fluxes, and global warming potential under legume cover and non-cropping cover and explored understory vegetation, soil physicochemical properties, and microorganisms as potential drivers of vegetation and soil carbon storage, soil greenhouse gas fluxes, and global warming potential under *Camellia oleifera* forests on a karst steep slope in Guizhou, which represents typical karst regions worldwide. Specifically, the following hypotheses were tested under *Camellia oleifera* forests: (1) cropping cover elevates understory vegetation biomass carbon storage compared to bare conditions; (2) cropping cover enhances soil CO_2_ flux; and (3) cropping cover reduces soil inorganic carbon storage. By quantifying this three-dimensional carbon shift in the *Camellia oleifera* forest ecosystem, our work offers important insights for cover cropping management not only in China’s karst regions but also in other global ecosystems with soil inorganic carbon, contributing to climate-smart agroforestry practices.

## 2 Materials and methods

### 2.1 Study area, experimental design, and sampling

The research area (N25°13′58.06″, E106°09′5.72″, 850 m a.s.l.) is located in Wangmo County, on the southern Guizhou Plateau, China. It experiences a subtropical monsoon humid climate, with dry winters and wet summers, and is prone to droughts in spring and autumn, and hot, rainy summers, with an average annual precipitation of 1,237 mm, an average annual sunshine duration of 1,402 h, an average frost-free period of 340 days, and an average annual temperature of 19 °C. For the purpose of economic benefits and oil-food security, *Camellia oleifera* forests are widely planted on steep karst slopes, with either cropping cover or without cover (clean, bare) commonly occurring under these forests. To minimize the differences arising from climate and soil background conditions, we established eight legume cropping (alfalfa) covered plots (eight replicates; CC) and eight adjacent non-cropping covered plots (eight replicates; NC) in *Camellia oleifera* forests on the same karst steep slope (slope = 20°). The seeding rate of alfalfa was 1.5 g/m^2^. Weeds were manually removed from both CC and NC plots before sowing and during the seedling stage. Visually, the NC plots were bare and exhibited soil erosion and degradation.

After measuring soil temperature at 5 cm depth (Shi H. et al., 2024) using soil thermometers (Zhang et al., 2017) (Shenzhen Lixinda Electronic Technology Co., Ltd., China), and recording the number of understory vegetation species in each plot (50 cm × 50 cm), we cut the aboveground parts of the understory vegetation at ground level using separate stainless-steel scissors in early June 2025. Subsequently, static chambers (inner diameter 23 cm, height 30 cm) were installed at the center of each plot, and gas samples were collected using separate 50-ml syringes immediately after chamber closure and again after 1 h. Each static chamber was equipped with a 1,500-rpm fan with seven 9-cm-long blades to ensure internal air mixing. The extracted gas was stored in separate gas sampling bags (Ningbo Hongpu Experimental Technology Co., Ltd., China). A total of 37 topsoil cores (0–5 cm) per plot were collected using separate stainless-steel ring cutters (inner diameter 5 cm, height 5 cm). Among them, 10 soil cores were used to obtain vegetation roots. Vegetation roots were collected and washed manually to remove soil using a 2-mm sieve and tweezers. The aboveground biomass of understory vegetation and vegetation roots was oven-dried at 105 °C for enzyme deactivation and then at 65 °C to constant weight using a precision drying oven (BPG-9140A, Shanghai Yiheng Scientific Instrument Co., Ltd., China), and recorded as understory vegetation aboveground biomass and vegetation root biomass, respectively. One intact soil core was used to determine soil capillary porosity, non-capillary porosity, and bulk density. The remaining 26 soil cores were homogenized, passed through a 2-mm sieve (Ye et al., 2022), and divided into three subsamples. One subsample was used to determine soil physicochemical properties, one was used to determine soil enzyme activity, and one was used for DNA extraction to determine soil bacterial and fungal communities. Sterile medical gloves (Yiwu Yintongmei Medical Technology Co., Ltd., China) were used throughout sampling to avoid cross-contamination.

### 2.2 Measurement of carbon storage, greenhouse gas fluxes, and global warming potential

Understory vegetation aboveground biomass and vegetation root biomass carbon content and soil organic carbon content were determined using the potassium dichromate–concentrated sulfuric acid external heating method (Lu L. et al., 2025). Understory vegetation aboveground biomass and vegetation root biomass carbon storage (g/m^2^) were calculated by multiplying the understory vegetation aboveground biomass and vegetation root biomass carbon content by the corresponding understory vegetation aboveground biomass and vegetation root biomass, respectively. Soil organic carbon content was determined using a TOC analyzer. Soil inorganic carbon content was determined using the volumetric titration method. Soil organic and inorganic carbon storage (g/m^2^) was calculated by multiplying soil organic and inorganic carbon content by soil bulk density and the sampling depth (5 cm) of the ring cutters. The concentrations of CO_2_, CH_4_, and N_2_O were determined within 2 weeks using a gas chromatograph (Agilent 7890B, Agilent Technologies, USA). CO_2_, CH_4_, and N_2_O fluxes (mg/m^2^/h) were calculated using the formula reported by Tarin et al. (2021), the parameters reported by Mon et al. (2024), and gas densities of 1.98, 0.717, and 1.97 mg/m^3^ for CO_2_, CH_4_, and N_2_O, respectively, under standard conditions. Global warming potential (kg CO_2_-equivalent/ha,100-year time scale) was calculated as CO_2_, 27.9 × CH_4_, and 273 × N_2_O (Ansari et al., 2023).

### 2.3 Determination of other soil physical and chemical properties and extracellular enzyme activity

Soil bulk density, capillary porosity, non-capillary porosity, and water content were determined by the oven-drying method. Soil pH was determined using potentiometry. Soil total nitrogen was determined using Kjeldahl digestion and a flow analyzer. Soil ammonium and nitrate nitrogen were determined using the potassium chloride extraction method and an autoanalyzer. Soil total phosphorus was determined using NaOH fusion and molybdenum–antimony colorimetry. Soil available phosphorus was determined using the double-acid extraction–molybdenum–antimony colorimetric method. Soil total and available potassium were determined using flame photometry. Soil total calcium and magnesium were determined using hydrochloric acid–nitric acid–perchloric acid digestion. Soil dissolved organic carbon was determined using the water extraction method. Microbial biomass carbon was determined using chloroform fumigation and the potassium dichromate–concentrated sulfuric acid external heating method. Microbial biomass nitrogen and phosphorus were determined using chloroform fumigation–potassium sulfate extraction and fumigation–extraction with UV spectrophotometry, respectively, and were used to estimate microbial carbon use efficiency *via* the R function “MicrobUIQ”^1^. POX (polyphenol oxidase), PER (peroxidase), and sucrase activities were measured using a spectrophotometer. βGC (β-1,4-glucosidase), CBH (cellobiohydrolase), NAG (β-1,4-N-acetylglucosaminidase), βX (β-1,4-xylosidase), *α*G (α-1,4-glucosidase), LAP (leucine aminopeptidase), and ACP (acid phosphatase) activities were measured using a fluorescence-based method. Enzyme vector analysis was used to assess carbon and phosphorus limitation, with vector angles >45° indicating phosphorus limitation in this study. This vector analysis was conducted using an R function (Ding and Wang, 2021) with the parameter “trans = 1” (Ding et al., 2023). Soil organic carbon decomposition was estimated using a microbial enzyme allocation model (Hill et al., 2014). Soil carbon quality was estimated using an enzyme-based lignocellulose index (Hill et al., 2014).

### 2.4 Amplification, sequencing of soil bacteria and fungi, and processing of sequencing data

Soil DNA was extracted using commercial DNA isolation kits (Lu L. et al., 2025). The bacterial 16S rRNA gene (V3–V4 region) was amplified using primers 341F and 806R (Chen et al., 2022). The fungal ITS1 region was amplified using primers 1737F and 2043R (Li H. et al., 2023). Sequencing was performed using the NovaSeq-PE250 platform. Paired-end reads were merged using FLASH v1.2.11^2^. High-quality clean tags were obtained using the fastp v0.23.1. Amplicon sequence variants (ASVs) were generated using DADA2 within QIIME2. Bacterial and fungal taxa were annotated using the SILVA and UNITE databases, respectively (Lu L. et al., 2025). Microbial diversity indices (observed ASVs and Pielou evenness) were calculated in QIIME2. Raw sequencing data for bacterial and fungal communities were deposited in the Figshare database^3^ with DOIs 10.6084/m9.figshare.30187921 and 10.6084/m9.figshare.30187990, respectively.

### 2.5 Soil bacterial and fungal composition, niche, life-history strategies, assembly mechanisms, and networks

Bacterial and fungal community composition differences were revealed using ANOSIM, ADONIS, and the multi-response permutation procedure (MRPP) with 999 permutations in the R “vegan” package. The reciprocal of the average variation degree (Lu J. et al., 2025) was used to represent bacterial and fungal community (composition and relative abundance) stability (Long et al., 2025). Bacterial and fungal niche breadth and overlap were estimated using the “spaa” package^4^ (Wang et al., 2022). Life-history strategies were annotated using the R function “YAS”^5^. Resource acquisition strategists catalyze decomposition and soil carbon loss; however, growth potential strategists may transform substrates into microbial biomass that contributes to organic carbon, whereas stress tolerance strategists may increase investment in maintenance and reduce biomass growth yield (Malik et al., 2020). Therefore, changes in life-history strategies may affect soil carbon dynamics (Ning et al., 2023; Liu et al., 2025). Bacterial and fungal community assembly processes were determined using the β-nearest taxon index (βNTI) and the Raup–Crick metric (RCbray) based on null models (Zhou and Ning, 2017; Ding et al., 2024) by applying the R “picante” package (Qiao et al., 2021) with 10,000 randomizations. βNTI < −2 and βNTI > 2 suggested homogeneous selection and variable selection, respectively (Qiao et al., 2021; Chen et al., 2022). |βNTI| < 2 with RCbray < −0.95 and |βNTI| < 2 with RCbray > + 0.95 suggested homogenizing dispersal and dispersal limitation, respectively (Qiao et al., 2021). |βNTI| < 2 with |RCbray| < 0.95 suggested drift (Ding et al., 2024). Bacterial and fungal networks were established using Spearman correlation analysis in the R “WGCNA” package and “igraph” packages, with ASVs that co-occurred in at least 25% of the total samples and with a total relative abundance of at least 0.0001. A correlation coefficient > 0.8 and *p* < 0.01 were considered to indicate potential interactions between microbiota (Ding et al., 2023). Furthermore, higher numbers of nodes and edges, higher average degree, connectance, and clustering coefficient, but lower average path length and diameter (the inverse of average path length and diameter), suggested greater microbial network complexity (Long et al., 2025). Network robustness analysis was used to assess bacterial and fungal network stability, and smaller declines in network natural connectivity with an increasing proportion of removed edges or nodes indicated greater stability (Ding et al., 2023). The decline in natural connectivity at 100% removal was used in the subsequent analysis.

### 2.6 Data analysis

After using the R function “nh.test”^6^ (Ding and Wang, 2021) to assess whether the data conformed to normality and homogeneity of variance, *t*-tests and Wilcoxon tests were used to assess the significance of differences between cropping cover and non-cropping cover for data that did and did not conform, respectively. Spearman correlation analysis was used to determine the relationships between carbon storage and greenhouse gas fluxes and plant, soil, and microbiota using the R “corrplot” package, and the results were displayed using the “pheatmap” package (Chen et al., 2022; Zheng et al., 2024). Redundancy analysis–based hierarchical partitioning with 10,000 permutations was used to separate the individual effects (i.e., contributions) of plant, soil, and microbial driving forces on cropping cover–induced variations in carbon storage and greenhouse gas fluxes by applying the R “rdacca.hp” package (Lai et al., 2022).

## 3 Results

### 3.1 Cropping cover altered carbon storage and greenhouse gas fluxes

**Figure 1:**
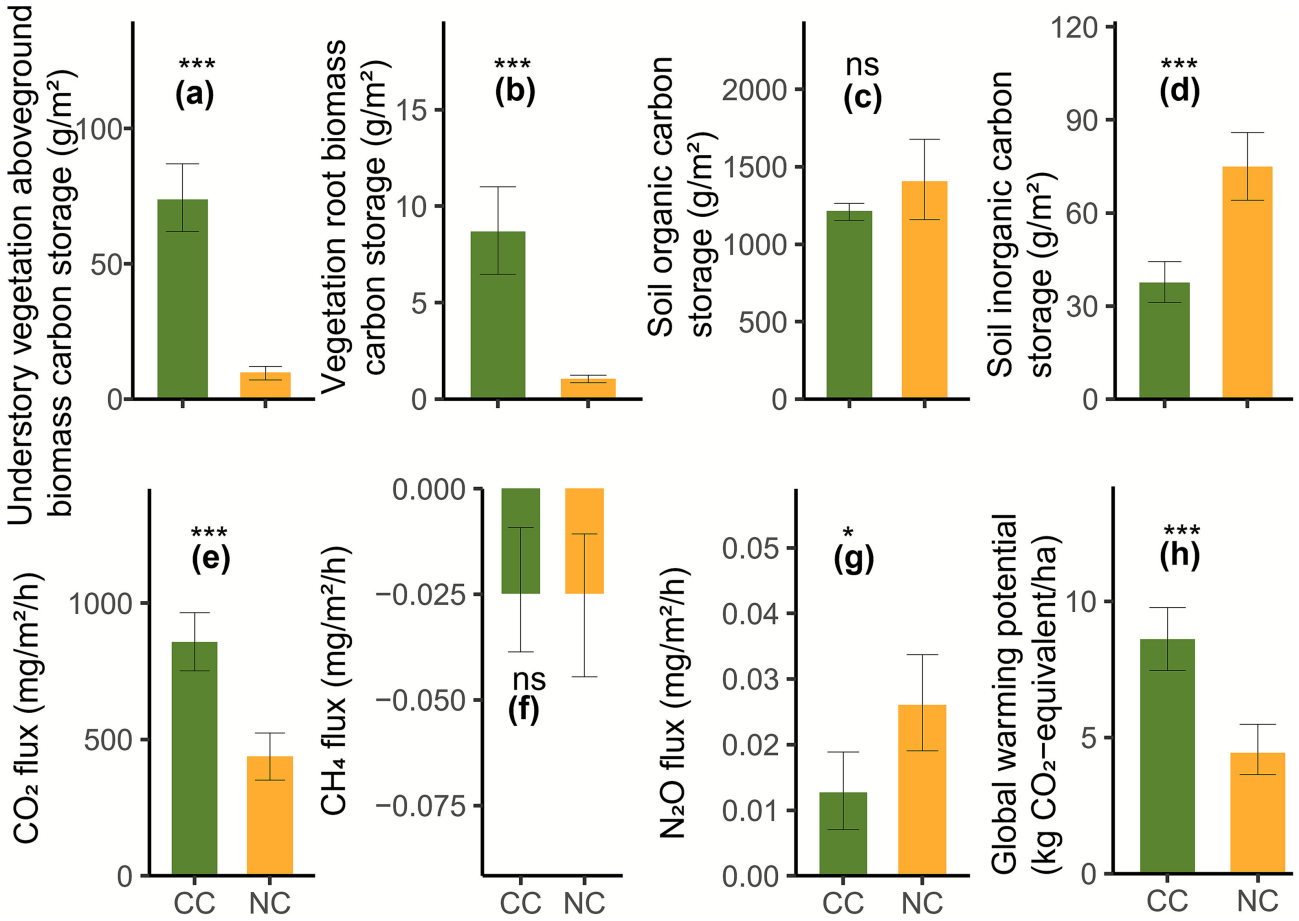
Understory vegetation aboveground **(a)**, vegetation root **(b)** biomass carbon storage, soil organic **(c)** and inorganic **(d)** carbon storage, soil CO_2_ **(e)**, CH_4_ **(f)**, N_2_O **(g)**, and global warming potential **(h)** under cropping cover (CC) and non-cropping cover (NC).

**Figure 2:**
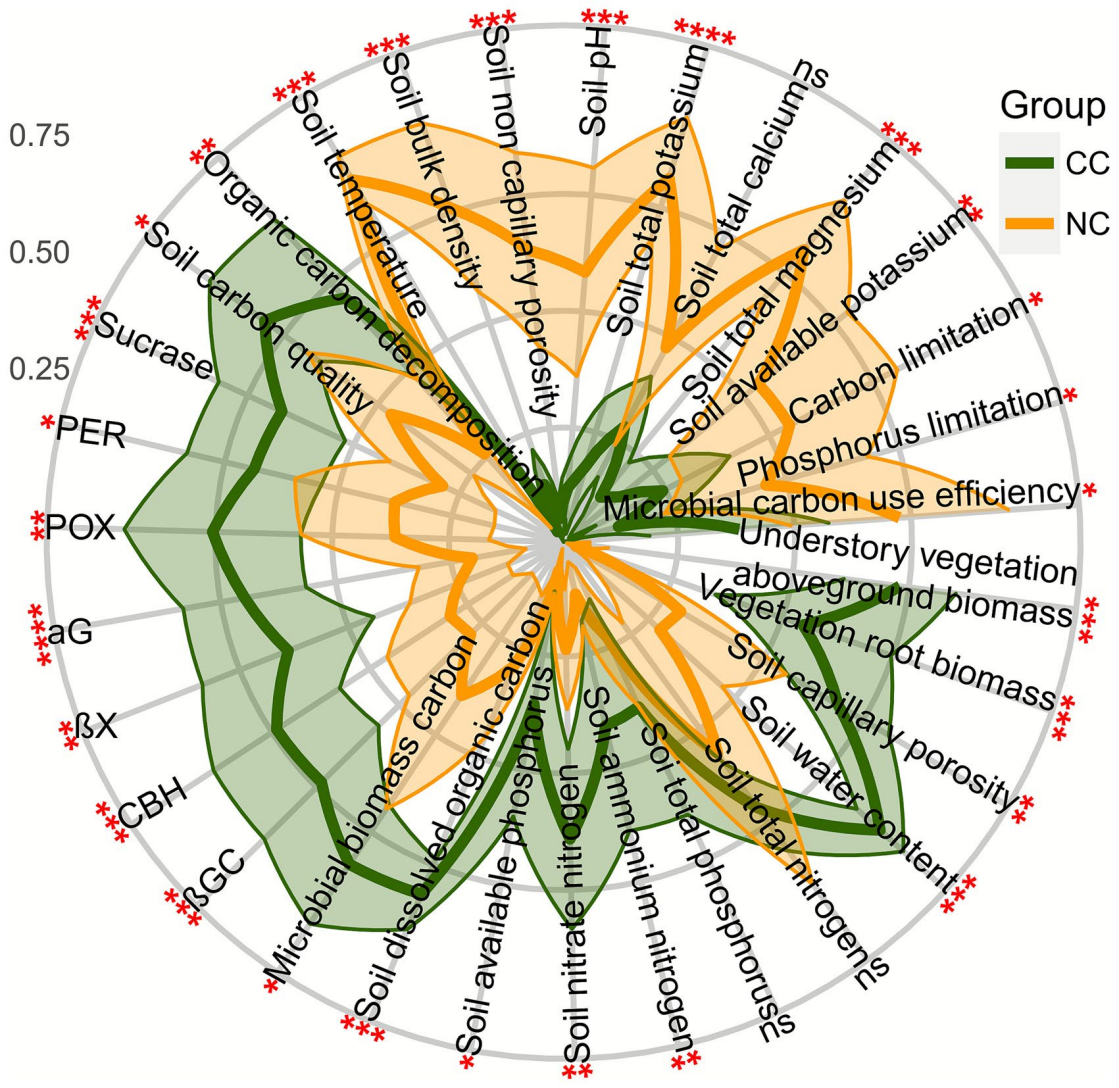
Differences in vegetation and soil physicochemical properties and carbon-related enzyme activities under cropping cover (CC) and non-cropping cover (NC). The shadow represents the 95% confidence interval. NS, no significant difference; *, *p* < 0.05; **, *p* < 0.01; ***, *p* < 0.001.

Cropping cover significantly modified carbon storage, greenhouse gas fluxes, and soil physical and chemical properties. Specifically, cropping cover had significantly higher understory vegetation aboveground (Figure 1a) and vegetation root (Figure 1b) biomass carbon storage than non-cropping cover (Wilcoxon test, *p* = 0.0002). However, it had significantly lower soil inorganic carbon storage than non-cropping cover (*t*-test, *p* = 0.0003; Figure 1d), although there was no significant difference in soil organic carbon storage (Wilcoxon test, *p* = 0.5100; Figure 1c). The negative CH_4_ flux indicated the extensive uptake of CH_4_ across the studied land; however, no significant difference in CH_4_ uptake was observed (*t*-test, *p* = 1; Figure 1f). Cropping cover had significantly higher CO_2_ flux than non-cropping cover (*t*-test, *p* = 0.0002; Figure 1e); however, it had significantly lower N_2_O flux than non-cropping cover (*t*-test, *p* = 0.0210; Figure 1g). Consequently, cropping cover had significantly higher global warming potential than non-cropping cover (*t*-test, *p* = 0.0002; Figure 1h). In addition, there were no significant differences in total nitrogen (Wilcoxon test, *p* = 0.9600), total phosphorus (*t*-test, *p* = 0.2200), or total calcium (*t*-test, *p* = 0.1200). However, cropping cover had significantly higher understory vegetation aboveground biomass (Wilcoxon test, *p* = 0.0002), vegetation root biomass (Wilcoxon test, *p* = 0.0009), soil capillary porosity (*t*-test, *p* = 0.0097), soil water content (Wilcoxon test, *p* = 0.0002), ammonium nitrogen (Wilcoxon test, *p* = 0.0019), nitrate nitrogen (*t*-test, *p* = 0.0013), available phosphorus (Wilcoxon test, *p* = 0.0104), dissolved organic carbon (Wilcoxon test, *p* = 0.0002), microbial biomass carbon (Wilcoxon test, *p* = 0.0104), βGC (*t*-test, *p* = 0.0009), CBH (*t*-test, *p* = 0.0004), βX (*t*-test, *p* = 0.0018), αG (*t*-test, *p* = 0.0000), POX (*t*-test, *p* = 0.0048), PER (*t*-test, *p* = 0.0169), sucrase (*t*-test, *p* = 0.0004), carbon quality (*t*-test, *p* = 0.0110), and organic carbon decomposition (*t*-test, *p* = 0.0043) than non-cropping cover. In contrast, cropping cover had significantly lower soil temperature (Wilcoxon test, *p* = 0.0008), bulk density (Wilcoxon test, *p* = 0.0003), non-capillary porosity (Wilcoxon test, *p* = 0.0009), pH (Wilcoxon test, *p* = 0.0009), total potassium (*t*-test, *p* = 0.0000), available potassium (Wilcoxon test, *p* = 0.0047), total magnesium (Wilcoxon test, *p* = 0.0002), carbon limitation (*t*-test, *p* = 0.0360), phosphorus limitation (Wilcoxon test, *p* = 0.0104), and microbial carbon use efficiency (*t*-test, *p* = 0.0180) than non-cropping cover (Figure 2).

### 3.2 Cropping cover reshaped understory vegetation species richness and soil microbial diversity

**Figure 3:**
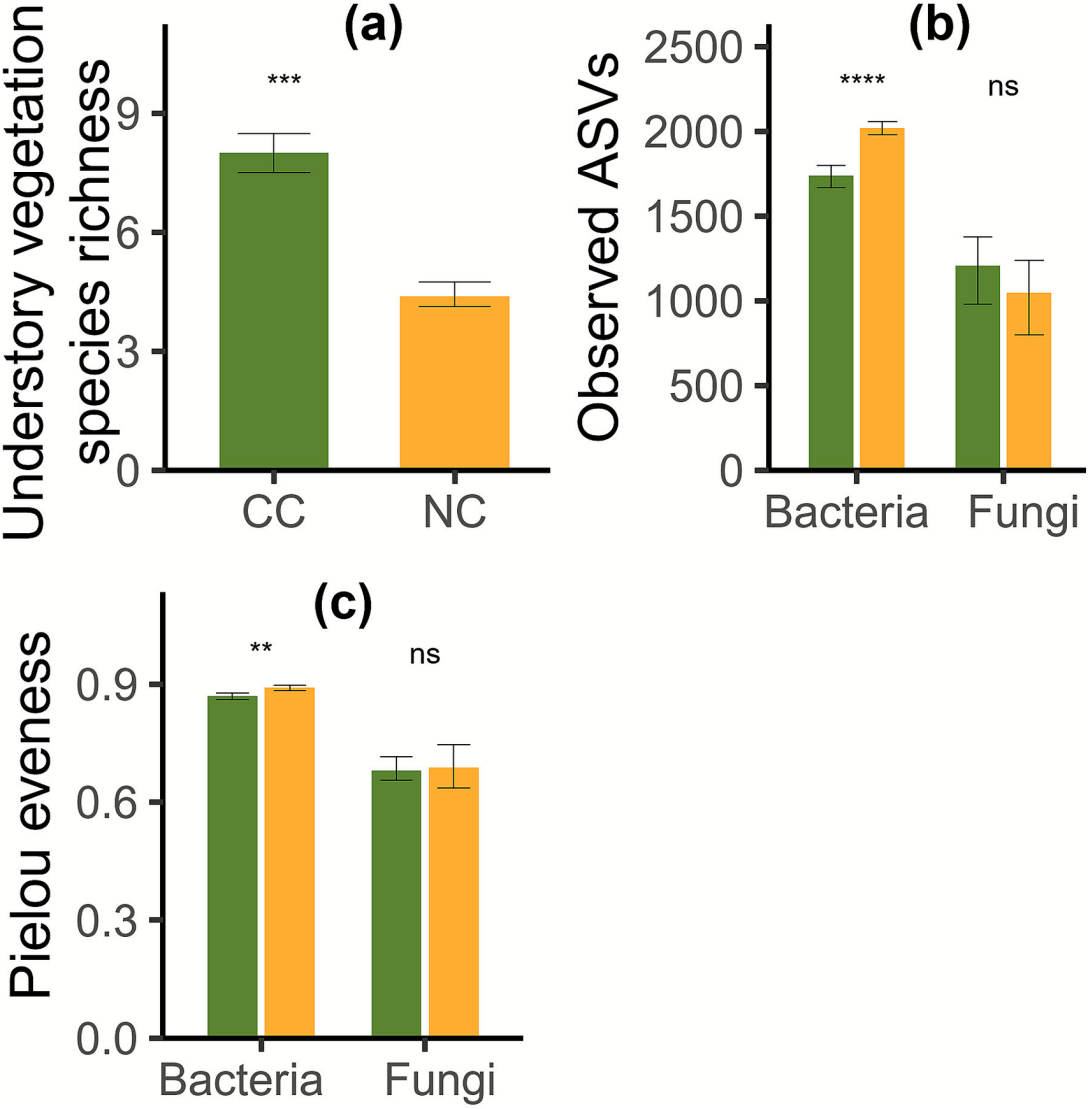
Understory vegetation species richness **(a)**, soil microbial observed ASVs **(b)**, and Pielou evenness **(c)** under cropping cover (CC) and non-cropping cover (NC). ns, No significant difference; **, *p* < 0.01; ***, *p* < 0.001; ****, *p* < 0.0001.

Cropping cover had significantly higher understory vegetation species richness than non-cropping cover (Wilcoxon test, *p* = 0.0104). However, it had significantly lower soil bacterial observed ASVs (*t*-test, *p* = 0.0000) and Pielou evenness (*t*-test, *p* = 0.0027) than non-cropping cover, whereas no significant difference in soil fungal diversity was observed (Wilcoxon test, *p* = 0.3700–1.0000; Figure 3).

### 3.3 Cropping cover reshaped soil microbial composition, niche, assembly mechanisms, life-history strategies, and microbial networks

**Figure 4:**
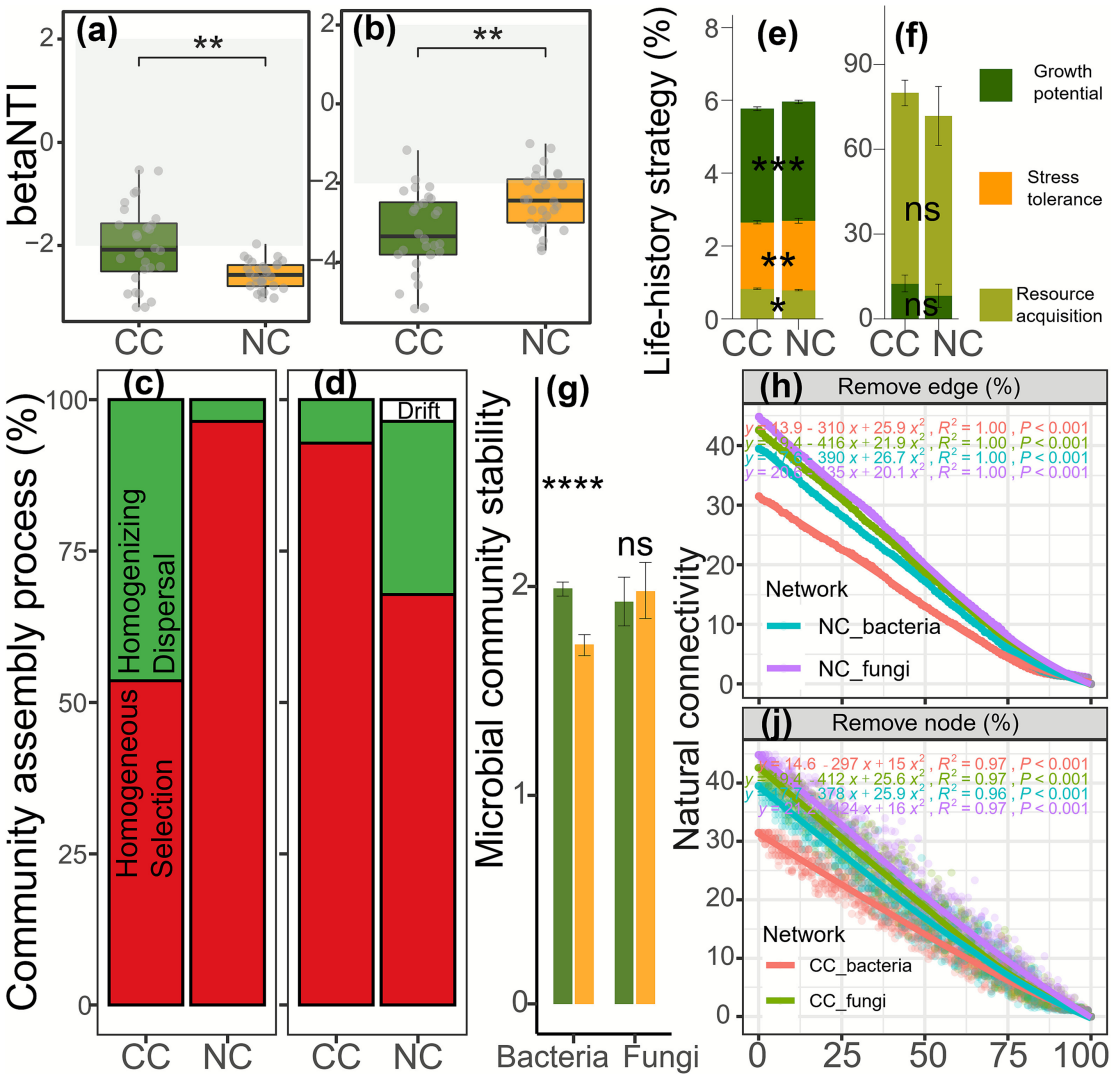
Soil bacterial **(a)** and fungal **(b)** βNTI, bacterial **(c)** and fungal **(d)** community assembly processes, bacterial **(e)** and fungal **(f)** life-history strategies, soil microbial community stability **(g)**, and network stability **(i and j)** under cropping cover (CC) and non-cropping cover (NC). NS, no significant difference; *, *p* < 0.05; **, *p* < 0.01; ***, *p* < 0.001; ****, *p* < 0.0001.

Cropping cover significantly reshaped soil microbial composition, niche breadth, assembly mechanisms, life-history strategies, and microbial networks. Specifically, ADONIS, ANOSIM, and MRPP indicated significant differences in bacterial (*p* = 0.0010) and fungal (*p* = 0.0010) community composition between cropping cover and non-cropping cover (Supplementary Table S1). Bacteria under cropping cover had significantly higher niche width than under non-cropping cover (Wilcoxon test, *p* < 0.0000), whereas fungi under cropping cover had significantly lower niche width than under non-cropping cover (Wilcoxon test, *p* < 0.0000; Supplementary Figure S1a). Both bacteria and fungi under cropping cover had significantly lower niche overlap than under non-cropping cover (Wilcoxon test, *p* < 0.0000; Supplementary Figure S1b). Furthermore, βNTI differed significantly for bacteria (Wilcoxon test, *p* = 0.0033) and fungi (*t*-test, *p* = 0.0011) between cropping cover and non-cropping cover. Bacteria under cropping cover exhibited higher homogenizing dispersal but lower homogeneous selection, whereas fungi under non-cropping cover exhibited higher drift and homogenizing dispersal but lower homogeneous selection (Figures 4a–d), indicating that cropping cover reshaped soil microbial assembly mechanisms. Moreover, bacteria under cropping cover had significantly lower growth potential (*t*-test, *p* = 0.0002) and stress tolerance (*t*-test, *p* = 0.0210) but higher resource acquisition (*t*-test, *p* = 0.0048) than under non-cropping cover. In contrast, fungi under cropping cover had no significant differences in growth potential (Wilcoxon test, *p* = 0.9600) and resource acquisition (*t*-test, *p* = 0.0640; Figures 4e,f). Microbial community stability analysis showed that soil bacterial community stability under cropping cover was significantly higher than under non-cropping cover (*t*-test, *p* = 0.0000; Figure 4g). Lower values of inverse bacterial diameter, inverse bacterial average path length, bacterial clustering coefficient, inverse fungal average path length, and fungal clustering coefficient indicated lower network complexity. The significantly lower values of these network metrics under cropping cover compared with non-cropping cover (Wilcoxon or *t*-test, *p* = 0.0002–0.0321; Supplementary Figure S1c) indicated reduced bacterial and fungal network complexity under cropping cover. Robustness analysis further showed that bacterial and fungal network stability under cropping cover was higher than that under non-cropping cover (Figures 4h,i).

### 3.4 Relationships between carbon storage, greenhouse gas fluxes, and plant, soil, and microbiota

**Figure 5:**
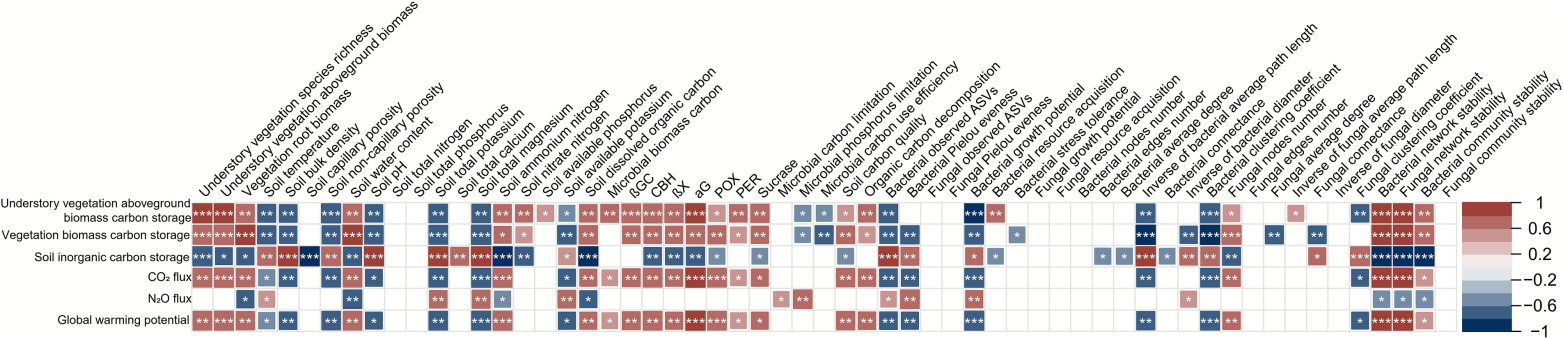
Spearman correlations of vegetation and soil carbon storage, greenhouse gas fluxes, and global warming potential with driving factors. *, *p* < 0.05; **, *p* < 0.01; ***, *p* < 0.001.

Carbon storage and greenhouse gas fluxes were strongly associated with plants, soil, and microbiota variables. Specifically, understory vegetation aboveground biomass carbon storage, vegetation root biomass carbon storage, CO_2_ flux, and global warming potential were positively correlated with understory vegetation species richness, aboveground biomass, vegetation root biomass, soil water content, ammonium nitrogen, dissolved organic carbon, CBH, ßX, αG, POX, sucrase, carbon quality, fungal node number, bacterial network stability, fungal network stability, and bacterial community stability (Spearman r = 0.5600–1.0000, *p* = 0.0000–0.0350). These variables were negatively correlated with soil temperature, bulk density, non-capillary porosity, pH, total potassium, total magnesium, available potassium, bacterial observed ASVs, bacterial growth potential, inverse bacterial average path length, and bacterial clustering coefficient (Spearman r = −0.86 to −0.53, *p* = 0.0000–0.0338). In contrast, soil inorganic carbon storage exhibited the opposite relationships (Spearman *p* = 0.0000–0.0400). N_2_O flux was positively correlated with soil temperature, total potassium, total magnesium, available potassium, carbon limitation, phosphorus limitation, bacterial observed ASVs, bacterial Pielou evenness, bacterial growth potential, and inverse bacterial diameter (Spearman r = −0.86 to −0.53, *p* = 0.0000–0.0338), but negatively correlated with vegetation root biomass, soil water content, soil ammonium nitrogen, dissolved organic carbon, bacterial network stability, fungal network stability, and bacterial community stability (Spearman r = −0.86 to −0.53, *p* = 0.0000–0.0338; Figure 5).

Furthermore, understory vegetation had a small but significant contribution to variation in aboveground biomass carbon storage (plant, *p* = 0.0004; soil, *p* = 0.8800; microbiota, *p* = 1.0000; Supplementary Figure S2a). The contribution of plant biomass (54.97%, *p* = 0.0009) exceeded that of plant richness (41.24%, *p* = 0.0008), and the contribution of understory vegetation aboveground biomass (71.88%, *p* = 0.0002) exceeded that of vegetation root biomass (23.71%, *p* = 0.0572). Understory vegetation also contributed significantly to variation in vegetation root biomass carbon storage (plant, *p* = 0.0001; soil, *p* = 0.8300; microbiota, *p* = 1.0000; Supplementary Figure S2b), with greater contributions from understory vegetation biomass (66.59%, *p* = 0.0004) than from plant richness (33.34%, *p* = 0.0025), and from vegetation root biomass (69.07%, *p* = 0.0002) than from understory vegetation aboveground biomass (30.84%, *p* = 0.0217). Understory vegetation also had a significant contribution to variation in soil inorganic carbon storage (plant, *p* = 0.0136; soil, *p* = 0.8100; microbiota, *p* = 1.0000; Supplementary Figure S2c), with a greater contribution from understory vegetation richness (36.36%, *p* = 0.0021) than from plant biomass (29.48%, *p* = 0.0714). Similar to patterns observed for understory vegetation aboveground biomass carbon storage, understory vegetation made a small but significant contribution to variation in soil CO_2_ flux and global warming potential (plant, *p* = 0.0098–0.0084; soil, *p* = 0.0570–0.5800; microbiota, *p* = 1.0000; Supplementary Figures S2d,f). Contributions from understory vegetation biomass (38.16–38.29%, *p* = 0.0192–0.0211) exceeded those from understory vegetation richness (31.12–31.19%, *p* = 0.0061–0.0070), and contributions from understory vegetation aboveground biomass (41.61–41.63%, *p* = 0.0075–0.0087) exceeded those from vegetation root biomass (27.60–27.83%, *p* = 0.0301–0.0332).

## 4 Discussion

### 4.1 Cropping cover changed understory vegetation and soil physical and chemical properties

Previous studies have suggested that high runoff removes fertile topsoil, thereby reducing nutrient availability (Capri et al., 2023). However, cropping cover reduced soil runoff (Li et al., 2012; Duan et al., 2020), erosion (Tu et al., 2021; Chen et al., 2025), and associated N and P losses (Chen et al., 2021; Li L. et al., 2023; Shirale et al., 2024; Andersen et al., 2025). In this study, cropping cover visibly increased understory vegetation species richness (Chen et al., 2025), understory vegetation aboveground biomass, and vegetation root biomass. These changes are beneficial for maintaining and improving soil physical and chemical properties.

Cropping cover did improve soil physical properties, supporting previous findings (Wei et al., 2018). For instance, cropping cover increased soil capillary porosity (Żelazny and Licznar-Małańczuk, 2018). Karst regions experience severe soil erosion and water loss; however, cropping cover reduced soil water loss (Ding et al., 2021). Capillary pores function as “small reservoirs” within the soil, retaining water primarily through capillary forces. In contrast, non-capillary pores act as “ventilation paths” and preferential flow paths in the soil, facilitating air circulation and water infiltration. The increased soil capillary porosity (Spearman r = 0.57, *p* = 0.0212) and reduced non-capillary porosity (Spearman r = −0.87, *p* = 0.0000) favor soil water conservation and contribute to higher soil water content, consistent with previous findings (Liu et al., 2015). Reduced soil water evaporation under cropping cover (Ding et al., 2021) further contributes to increased soil water content.

Furthermore, cropping cover improved soil chemical properties (Wei et al., 2018; Li et al., 2022; Wang et al., 2023; Dong et al., 2024; Wang et al., 2024; Chen et al., 2025), carbon quality, and enzyme activities (Tang et al., 2022; Shi C. et al., 2024) (Figure 2), alleviating carbon and phosphorus limitations and enhancing microbial biomass carbon (Wei et al., 2018; Xiang et al., 2023; Chen et al., 2025) and organic carbon decomposition (Figure 2). Although some studies reported that understory vegetation depletes soil nutrients (Wang R. et al., 2020) and water (Żelazny and Licznar-Małańczuk, 2018), our findings align with studies demonstrating improved soil fertility under cropping cover (Xiang et al., 2022; Xiao et al., 2022; Pedraza and Gonzalez-Andujar, 2025). Soil microbial carbon and phosphorus limitations have been reported in *Camellia oleifera* forests, consistent with previous studies (Qiao et al., 2021). In contrast to peanut cover, which alleviated microbial nitrogen limitation but exacerbated phosphorus limitation (Xu et al., 2023), alfalfa cover alleviated both soil microbial carbon and phosphorus limitation (Figure 2). Higher investment in microbial resource acquisition (Figures 2, 4; e.g., carbon acquisition enzymes) corresponded with reduced microbial carbon use efficiency under cropping cover. In addition, lower soil pH under cropping cover (Tang et al., 2022) can be attributed to enhanced nitrogen availability associated with legumes (Shirale et al., 2024; Wang et al., 2024; Zheng et al., 2024). Higher total potassium (Zang et al., 2025), available potassium (Wang R. et al., 2020; Wang et al., 2024), and total magnesium under non-cropping cover can be attributed to mineral weathering following surface soil erosion. However, alfalfa cultivation has been reported to reduce soil available potassium through plant uptake (Ding et al., 2021). Together, these processes explain the lower available potassium observed under alfalfa cover relative to non-cropping cover.

Overall, legume cropping cover is supported from the perspective of improving understory vegetation and soil physical and chemical properties.

### 4.2 Cropping cover shifted soil microbial diversity, assembly mechanisms, life-history strategies, and microbial networks

As a result of improving understory vegetation and soil physicochemical properties, soil microbial communities were affected (Laurent et al., 2008; Chen et al., 2014; Wan and He, 2021). Contrary to previously reported increases in soil microbial diversity under cropping cover (Xiao et al., 2022; Li H. et al., 2023; Xiang et al., 2023; Zhuo et al., 2025), cropping cover in this study reduced soil bacterial diversity (Bajiu et al., 2024), including richness (Li et al., 2022) and Pielou evenness, compared with non-cropping cover. Two mechanisms may explain this decline. First, soil pH is one of the most important determinants of soil bacterial communities in *Camellia oleifera* forests (Qiao et al., 2021; Chen et al., 2022; Ye et al., 2022; Liu et al., 2024; Lv et al., 2024; Qiao et al., 2024; Lu J. et al., 2025), and the cropping cover–induced decline in soil pH (Tang et al., 2022) was significantly associated with reduced soil bacterial diversity (Spearman r = 0.6–80.87, *p* = 0.0000–0.0040). Second, cropping cover supplied a resource-rich habitat with higher labile carbon (e.g., dissolved organic carbon) and nutrient availability (Rodriguez-Ramos et al., 2022) (Figure 2). This shift reduced homogeneous selection but enhanced homogenizing dispersal of soil bacteria (Figure 4c), accompanied by lower stress tolerance and greater investment in resource acquisition strategies (Figure 4e). Such changes may have contributed to a simplified microbial community structure and reduced network complexity. These patterns have not previously been reported in crop-covered *Camellia oleifera* forests. Recent studies have shown that cropping cover enhanced soil microbial network stability in soybean cover-crop rotation systems (Guo et al., 2024) and that *Camellia oleifera* varieties influence soil microbial community stability (Lu J. et al., 2025). In contrast, the present study demonstrates, for the first time within *Camellia oleifera* forest ecosystems, that cover cropping enhanced the stability of soil bacterial communities as well as soil bacterial and fungal networks. Nevertheless, the underlying mechanisms warrant further investigation. Overall, from the perspective of improving microbial community stability and network stability, this study supports the use of cropping cover.

### 4.3 Cropping cover induced a vegetation–soil carbon trade-off

Previous studies have mainly demonstrated the positive effect of cropping cover on soil organic carbon (Wei et al., 2018; Xiang et al., 2022; Shi H. et al., 2024; Xiang et al., 2024), but this study supports a neutral role (Chen et al., 2025). Cropping cover–induced increases in understory vegetation species richness (Figure 3a), understory aboveground (Figure 2), and vegetation root (Figure 2) biomass were significantly and positively correlated with understory vegetation aboveground (Figure 1a; Supplementary Figure S2a) and vegetation root (Figure 1b; Supplementary Figure S2b) biomass carbon storage, respectively (Spearman r = 0.70–0.99, *p* = 0.0000–0.0025), supporting previous viewpoints (Deng et al., 2023; Dang et al., 2024; Ullah et al., 2024; Li et al., 2025). Previous studies found vegetation species richness enhanced soil organic carbon storage (Chen et al., 2018; Dang et al., 2024). However, in this study, cropping cover–induced increases in understory vegetation species richness significantly and negatively correlated with soil inorganic carbon storage (Spearman r = −0.79, *p* = 0.0002; Figure 1d; Supplementary Figure S2c), emphasizing the dilemma of co-management of biodiversity and soil inorganic carbon storage. A previous study identified a trade-off between plant carbon storage and soil organic carbon storage (Terrer et al., 2021). In contrast, this study demonstrated for the first time a trade-off between understory vegetation aboveground (Spearman r = −0.69, *p* = 0.0030) and vegetation root biomass carbon storage (Spearman r = −0.62, *p* = 0.0099) and soil inorganic carbon storage. Furthermore, in the study by Terrer et al. (2021), the trade-off between plant carbon storage and soil organic carbon storage was attributed to plant nutrient acquisition, whereby biomass augmentation through soil nutrient mining resulted in diminished soil organic carbon storage. However, in our study, the trade-off between plant carbon storage and soil inorganic carbon storage can also be explained by cropping cover–induced changes in soil pH and soil water and magnesium contents. Cropping cover–induced declines in soil pH (Tang et al., 2022) (Spearman r = 0.81, *p* = 0.0002) and increases in soil water content (Spearman r = −0.70, *p* = 0.0024) may promote the dissolution and release of soil inorganic carbon (Figure 5). Plant growth also relied on the absorption and consumption of magnesium (Spearman r = −0.80 to −0.72, *p* = 0.0002–0.0015) produced by the dissolution of inorganic carbonates, which was associated with reduced soil inorganic carbon storage (Spearman r = 0.81, *p* = 0.0001; Figure 5). These findings suggest that in soils with inorganic carbon, particularly those dominated by inorganic carbon, the relationship between plant carbon storage and soil inorganic carbon storage should be carefully considered when conducting multi-storage management. Notably, the gain in understory vegetation carbon storage (71.51 g/m^2^) exceeded the loss of soil inorganic carbon storage (37.36 g/m^2^). This supports the importance of understory carbon storage in the context of the global climate change mitigation potential (Dirnböck et al., 2020) and suggests that cropping cover could be recommended from the perspective of vegetation–soil carbon storage trade-offs.

### 4.4 Cropping cover increased global warming potential via enhanced CO₂ flux, despite reduced N₂O flux

The extensive absorption of CH_4_ indicated a CH_4_ sink across the studied land, supporting previous findings (Chen et al., 2020). Furthermore, the reduction in N₂O flux under cropping cover supported previous studies (Muhammad et al., 2019; Wang H. et al., 2020). CH_4_ uptake and reduced N₂O flux were beneficial for mitigating global greenhouse gas emissions and warming. Previous studies have shown that soil water content and temperature are key regulators of soil N_2_O emissions in karst soils (Chen et al., 2020). This study showed that the negative effect of elevated soil water content (individual effect = 29.96%, *p* = 0.0273; Spearman r = −0.72, *p* = 0.0017) was greater than the negative effect of decreased soil temperature (individual effect = 16.95%, *p* = 0.1117; Supplementary Figure S2e; Spearman r = 0.53, *p* = 0.0337) on soil N_2_O flux. In addition, the negative effect of decreased bacterial Pielou evenness (individual effect = 26.70%, *p* = 0.0374; Spearman r = 0.74, *p* = 0.0471) was greater than that of decreased bacterial observed ASVs (individual effect = 13.83%, *p* = 0.1567; Spearman r = 0.50, *p* = 0.0011). The negative effect of elevated microbial network stability (individual effect = 23.37%, *p* = 0.0429; Supplementary Figure S2e; Spearman r = −0.81, *p* = 0.0213) was also greater than that of reduced microbial network complexity (individual effect = 13.41%, *p* = 0.1541; Spearman r = 0.54, *p* = 0.0319). These negative effects help explain the reduction in N_2_O flux under cropping cover compared with non-cropping cover (Figure 1g).

However, cropping cover–induced increases in understory vegetation species richness (individual effect = 31.19%, *p* = 0.0061) and biomass (individual effect = 38.29%, *p* = 0.0192), particularly the increase in understory vegetation aboveground biomass (Figure 1a, individual effect = 41.61%, *p* = 0.0075; Supplementary Figure S2d), significantly enhanced CO_2_ flux (Figure 1e; Spearman r = 0.77, *p* = 0.0005). The enhancing effects of cropping cover (Muhammad et al., 2019) and vegetation species richness (Dang et al., 2024) on CO_2_ flux are consistent with global studies. Two mechanisms may explain this enhancement. First, cropping cover can reduce soil organic carbon loss by weakening erosive forces from runoff and rainfall (Zheng et al., 2021). Second, increased understory vegetation aboveground (Figure 1a) and root biomass (Figure 1b) enhanced organic matter inputs via root exudates and residue decomposition (Chen et al., 2025), improving nutrient availability for soil microbiota (Rodriguez-Ramos et al., 2022) (Spearman r = 0.54–0.71, *p* = 0.0019–0.0293). In particular, increases in vegetation biomass (Wu et al., 2021) (Spearman r = 0.51–0.53, *p* = 0.0338–0.0445) and available phosphorus (Xue et al., 2023; Lv et al., 2025) (Spearman r = −0.51, *p* = 0.03741) probably alleviated microbial phosphorus limitation (Figure 2). Enhanced nutrient supply supported higher microbial biomass carbon (Shi H. et al., 2024) (Figure 2; Spearman r = 0.54–0.64, *p* = 0.0074–0.0315), thereby accelerating soil carbon mineralization (Wei et al., 2018) and organic carbon decomposition (Figure 2; Spearman r = 0.58–0.87, *p* = 0.0000–0.0187), ultimately increasing CO_2_ flux (Figure 2; Spearman r = 0.52–0.76, *p* = 0.0006–0.0387). Carbohydrate-degrading enzymes are crucial for plant litter decomposition (Zheng et al., 2018) and soil nutrient cycling (Wang Y. et al., 2020). Increases in CBH (Gu et al., 2025), βX (Wang Y. et al., 2020), αG, βGC (Sun et al., 2025), POX, PER (Duanyuan et al., 2023), and sucrase (Xue et al., 2023) (Figure 2) facilitated organic carbon decomposition (Spearman r = 0.56–0.94, *p* = 0.0235–0.0000). However, this decomposition did not reduce soil organic carbon storage (Spearman r = 0.09, *p* = 0.7535), likely due to continuous inputs from understory vegetation. In addition, the trade-off between CO_2_ flux and soil inorganic carbon storage (Spearman r = −0.76, *p* = 0.0007) suggests that a portion of emitted CO_2_ was derived from inorganic carbon dissolution.

Cropping cover increased global warming potential (Figure 1h), consistent with recent studies (Ansari et al., 2023; Shi H. et al., 2024). Cropping cover–induced increases in understory vegetation species richness (individual effect = 31.12%, *p* = 0.0070) and biomass (individual effect = 38.16%, *p* = 0.0211), especially the increase in understory vegetation aboveground biomass (Figure 1a; individual effect = 41.63%, *p* = 0.0087; Supplementary Figure S2f), were associated with significantly elevated global warming potential (Figure 1e; Spearman r = 0.77, *p* = 0.0005). As the contribution of soil microbiota to soil CO_2_ and N_2_O fluxes and global warming potential was 65–66% higher than the contribution of understory vegetation, we further divided the role of microbiota into microbial diversity, microbial life-history strategy, and microbial network and suggested that reduced microbial life-history strategy (that is, bacterial growth potential; Figure 4e) contributed to elevated global warming potential (individual effect = 20.02%, *p* = 0.0411; Spearman r = −0.76, *p* = 0.0006; Figures 4, 5). We further divided microbial diversity into richness and evenness and microbial network into microbial network complexity and network stability and found that reduced microbial richness (that is, bacterial observed ASVs, individual effect = 27.81%, *p* = 0.0341) exerted a greater effect than reduced microbial evenness (i.e., bacterial Pielou evenness; individual effect = 16.52%, *p* = 0.1203) in elevating global warming potential (Spearman r = −0.66 to −0.63, *p* = 0.0055–0.0092, Figures 4, 5). Enhanced microbial network stability (individual effect = 34.26%, *p* = 0.0030, Spearman r = 0.81, *p* = 0.0001, Figures 4, 5) exerted a greater role than reduced microbial network complexity (individual effect = 17.54%, *p* = 0.7147, Spearman r = −0.60 to −0.75, *p* = 0.0008–0.0134) and enhanced microbial (bacterial) community stability (individual effect = 16.84%, *p* = 0.0717, Spearman r = 0.57, *p* = 0.0210) in elevating global warming potential. Bacterial network stability (individual effect = 32.73%, *p* = 0.0209) exerted a positive role compared to fungal network stability (individual effect = 32.73%, *p* = 0.0207, Supplementary Figure S2f). Similar driving mechanisms also occurred in CO_2_ flux but not in N_2_O flux. These findings have not been reported previously, emphasizing the importance and necessity of this study.

Collectively, alfalfa cover may not be recommended in *Camellia oleifera* forests from the perspective of global greenhouse gas emissions reduction and mitigating global warming.

## 5 Conclusion

Introducing alfalfa as a cropping cover in *Camellia oleifera* forests on karst steep slopes creates a significant carbon trade-off, which substantiates our three hypotheses. Alfalfa cover is recommended for improving understory vegetation aboveground and vegetation root biomass carbon storage, soil physicochemical properties, and soil microbial community and network stability; however, it may not be recommended due to increased soil CO₂ flux and global warming potential, as well as reduced soil inorganic carbon storage. This study is the first to explicitly quantify this trade-off between enhanced vegetation carbon storage and the loss of soil inorganic carbon storage in this context. Therefore, management strategies aimed at maximizing carbon sequestration in karst agroforestry systems must carefully balance the promotion of understory biomass against the potential depletion of substantial inorganic carbon storage. Future practices should be designed to mitigate this trade-off to achieve genuine multi-carbon storage benefits.

## Data Availability

The data presented in the study are deposited in the figshare repository, accession number 10.6084/m9.figshare.30187921 and 10.6084/m9.figshare.30187990. Other data are provided in the Supplementary materials.

## Footnotes

1
https://github.com/dlltargeting/MicrobUIQ

2
http://ccb.jhu.edu/software/FLASH/

3
https://figshare.com

4
https://github.com/helixcn/spaa

5
https://github.com/dlltargeting/YAS

6
https://github.com/dlltargeting/nh.test

## References

[ref1] Andersen M. S. Engedal T. Bruun S. Jensen L. S. Hansen V. (2025). Emissions of N_2_O following field incorporation of leguminous and non-leguminous cover crops. Agric. Ecosyst. Environ. 379:109335. doi: 10.1016/j.agee.2024.109335

[ref2] Ansari J. Davis M. P. Anderson S. H. Eivazi F. Bardhan S. (2023). Greenhouse gas emissions from row crop, agroforestry, and forested land use systems in floodplain soils. Water Air Soil Pollut. 234:227. doi: 10.1007/s11270-023-06227-6

[ref3] Bajiu A. Gao K. Zeng G. He Y. (2024). Impact of intercropping five medicinal plants on soil nutrients, enzyme activity, and microbial community structure in Camellia oleifera plantations. Microorganisms 12:1616. doi: 10.3390/microorganisms12081616, 39203458 PMC11356553

[ref4] Capri C. Gatti M. Fiorini A. Ardenti F. Tabaglio V. Poni S. (2023). A comparative study of fifteen cover crop species for orchard soil management: water uptake, root density traits and soil aggregate stability. Sci. Rep. 13:721. doi: 10.1038/s41598-023-27915-7, 36639732 PMC9839681

[ref5] Chen L. Bao Y. He X. Yang J. Wu Q. Lv J. (2025). Nature-based accumulation of organic carbon and nitrogen in citrus orchard soil with grass coverage. Soil Tillage Res. 248:106419. doi: 10.1016/j.still.2024.106419

[ref6] Chen L. Mei L. Chen Y. Zhao Z. Xu Y. Zhang Z. . (2021). Effects of interplanting herbage on surface runoff associated with nitrogen and phosphorus losses in *Camellia oleifera* plantations. J. Nanjing For. Univ. 45, 127–134. doi: 10.12302/j.issn.1000-2006.202101035

[ref7] Chen S. Wang W. Xu W. Wang Y. Wan H. Chen D. . (2018). Plant diversity enhances productivity and soil carbon storage. Proc. Natl. Acad. Sci. 115, 4027–4032. doi: 10.1073/pnas.1700298114, 29666315 PMC5910804

[ref8] Chen Y. Wen X. Sun Y. Zhang J. Wu W. Liao Y. (2014). Mulching practices altered soil bacterial community structure and improved orchard productivity and apple quality after five growing seasons. Sci. Hortic. 172, 248–257. doi: 10.1016/j.scienta.2014.04.010

[ref9] Chen L. Wu L. Sun Q. Chen Y. Wang C. Lu s. (2022). Long-term *Camellia oleifera* cultivation influences the assembly process of soil bacteria in different soil aggregate particles. Land Degrad. Dev. 34, 441–452. doi: 10.1002/ldr.4470

[ref10] Chen P. Zhou M. Wang S. Luo W. Peng T. Zhu B. . (2020). Effects of afforestation on soil CH4 and N_2_O fluxes in a nsubtropical karst landscape. Sci. Total Environ. 705:135974. doi: 10.1016/j.scitotenv.2019.135974, 31841922

[ref11] Dang P. Zhang M. Chen X. Loreau M. Duffy J. E. Li X.e. . (2024). Plant diversity decreases greenhouse gas emissions by increasing soil and plant carbon storage in terrestrial ecosystems. Ecol. Lett. 27:14469. doi: 10.1111/ele.14469, 38990962

[ref12] Deng J. Fang S. Fang X. Jin Y. Kuang Y. Lin F. . (2023). Forest understory vegetation study: current status and future trends. For. Res. 3:6. doi: 10.48130/fr-2023-0006, 39526278 PMC11524240

[ref13] Ding L. Chen H. Wang M. Wang P. (2024). Shrub expansion raises both aboveground and underground multifunctionality on a subtropical plateau grassland: coupling multitrophic community assembly to multifunctionality and functional trade-off. Front. Microbiol. 14:9125. doi: 10.3389/fmicb.2023.1339125, 38274762 PMC10808678

[ref14] Ding L. Tian L. Li J. Zhang Y. Wang M. Wang P. (2023). Grazing lowers soil multifunctionality but boosts soil microbial network complexity and stability in a subtropical grassland of China. Front. Microbiol. 13:1027097. doi: 10.3389/fmicb.2022.1027097, 36687566 PMC9849757

[ref15] Ding L. Wang P. (2021). Afforestation suppresses soil nitrogen availability and soil multifunctionality on a subtropical grassland. Sci. Total Environ. 761:143663. doi: 10.1016/j.scitotenv.2020.143663, 33360134

[ref16] Ding T. Yan Z. Zhang W. Duan T. (2021). Green manure crops affected soil chemical properties and fungal diversity and community of apple orchard in the loess plateau of China. J. Soil Sci. Plant Nutr. 21, 1089–1102. doi: 10.1007/s42729-021-00424-0

[ref17] Dirnböck T. Kraus D. Grote R. Klatt S. Kobler J. Schindlbacher A. . (2020). Substantial understory contribution to the C sink of a European temperate mountain forest landscape. Landsc. Ecol. 35, 483–499. doi: 10.1007/s10980-019-00960-2, 32165789 PMC7045765

[ref18] Dong R. Hu W. Bu L. Cheng H. Liu G. (2024). Legume cover crops alter soil phosphorus availability and microbial community composition in mango orchards in karst areas. Agric. Ecosyst. Environ. 364:108906. doi: 10.1016/j.agee.2024.108906

[ref19] Duan J. Liu Y.-J. Yang J. Tang C.-J. Shi Z.-H. (2020). Role of groundcover management in controlling soil erosion under extreme rainfall in citrus orchards of southern China. J. Hydrol. 582:124290. doi: 10.1016/j.jhydrol.2019.124290

[ref20] Duanyuan H. Zhou T. He Z. Peng Y. Lei J. Dong J. . (2023). Effects of straw mulching on soil properties and enzyme activities of *Camellia oleifera*–*Cassia* intercropping agroforestry systems. Plants 12:3046. doi: 10.3390/plants12173046, 37687293 PMC10490048

[ref21] Fei Y. Lu R. Luo S. Luo S. Wei A. Zhou Y. . (2025). Application analysis of grass cultivation technology in orchard. Agric. Eng. 15, 80–84. doi: 10.19998/j.cnki.2095-1795.202501313

[ref22] Gu Y. Jiao J. Xu H. Chen Y. He X. Wu X. . (2025). Intercropping improves the yield by increasing nutrient metabolism capacity and crucial microbial abundance in root of Camellia oleifera in purple soil. Plant Physiol. Biochem. 219:9318. doi: 10.1016/j.plaphy.2024.109318, 39608339

[ref23] Guo Y. Wang H. Du L. Shi P. Du S. Xu Z. . (2024). Microbial communities mediate the effect of cover cropping on soil ecosystem functions under precipitation reduction in an agroecosystem. Sci. Total Environ. 947:174572. doi: 10.1016/j.scitotenv.2024.174572, 38986707

[ref24] Haq S. M. Waheed M. Darwish M. Siddiqui M. H. Goursi U. H. Kumar M. . (2024). Biodiversity and carbon stocks of the understory vegetation as indicators for forest health in the Zabarwan Mountain range, Indian Western Himalaya. Ecol. Indic. 159:111685. doi: 10.1016/j.ecolind.2024.111685

[ref25] Hill B. H. Elonen C. M. Jicha T. M. Kolka R. K. Lehto L. L. P. Sebestyen S. D. . (2014). Ecoenzymatic stoichiometry and microbial processing of organic matter in northern bogs and fens reveals a common P-limitation between peatland types. Biogeochemistry 120, 203–224. doi: 10.1007/s10533-014-9991-0

[ref26] Hoagland L. Carpenter-Boggs L. Granatstein D. Mazzola M. Smith J. Peryea F. . (2008). Orchard floor management effects on nitrogen fertility and soil biological activity in a newly established organic apple orchard. Biol. Fertil. Soils 45, 11–18. doi: 10.1007/s00374-008-0304-4

[ref27] Hu Y. Zhan P. Thomas B. W. Zhao J. Zhang X. Yan H. . (2022). Organic carbon and nitrogen accumulation in orchard soil with organic fertilization and cover crop management: a global meta-analysis. Sci. Total Environ. 852:158402. doi: 10.1016/j.scitotenv.2022.158402, 36055500

[ref28] Krach R. J. Daley C. A. Liles G. C. (2025). Climate smart management practices add value to mature organic almond production system. Front. Sustain. Food Syst. 9:898. doi: 10.3389/fsufs.2025.1527898

[ref29] Lai J. Zou Y. Zhang J. Peres-Neto P. R. (2022). Generalizing hierarchical and variation partitioning in multiple regression and canonical analyses using the rdacca.Hp R package. Methods Ecol. Evol. 13, 782–788. doi: 10.1111/2041-210x.13800

[ref30] Laurent A. S. Merwin I. A. Thies J. E. (2008). Long-term orchard groundcover management systems affect soil microbial communities and apple replant disease severity. Plant Soil 304, 209–225. doi: 10.1007/s11104-008-9541-4

[ref31] Li L. Chen P. Wang K. Zhang R. Yuan X. Ge L. . (2023). Gramineae-legumes mixed planting effectively reduces soil and nutrient loss in orchards. Agric. Water Manag. 289:108513. doi: 10.1016/j.agwat.2023.108513

[ref32] Li T. Wang Y. Kamran M. Chen X. Tan H. Long M. (2022). Effects of grass inter-planting on soil nutrients, enzyme activity, and bacterial community diversity in an apple orchard. Front. Plant Sci. 13:441. doi: 10.3389/fpls.2022.901143, 35837455 PMC9274827

[ref33] Li H. Wang X. Li Y. Hou Y. Zhao Z. Meng L. . (2023). Cover crops control weed and improve soil qualities in citrus orchard. J. Soil Sci. Plant Nutr. 23, 6827–6837. doi: 10.1007/s42729-023-01545-4

[ref34] Li J. Wang Z. Liu S. Qin C. Li Q. He X. . (2025). The universal but weak positive correlation between plant diversity and carbon storage: evidence from a global synthetic analysis. Land Degrad. Dev. 36, 3898–3915. doi: 10.1002/ldr.5606

[ref35] Li X. H. Yang J. Zhao C. Y. Wang B. (2012). Runoff and sediment from orchard terraces in southeastern China. Land Degrad. Dev. 25, 184–192. doi: 10.1002/ldr.1160

[ref36] Liu Y. Chen Z. Li L. Sun Y. Hu X. Zhang X. . (2025). Linking prokaryotic life-history strategies to soil organic carbon stability in semi-arid orchard with cover crops. Catena 252:108833. doi: 10.1016/j.catena.2025.108833

[ref37] Liu C. He Z. Chen Y. Xu Y. Tang W. Chen L. (2024). Effects of nitrogen deposition on the rhizosphere nitrogen-fixing bacterial community structure and assembly mechanisms in *Camellia oleifera* plantations. Front. Microbiol. 15:14724. doi: 10.3389/fmicb.2024.1414724, 38957615 PMC11217174

[ref38] Liu Y. Wang J. Fu F. Chu G. (2015). Effect of grass planting in orchard on soil ecological environment. J Yangzhou Univ 36, 110–113. doi: 10.16872/j.cnki.1671-4652.2015.04.023

[ref39] Long X. Li J. Liao X. Wang J. Zhang W. Wang K. . (2025). Stable soil biota network enhances soil multifunctionality in agroecosystems. Glob. Chang. Biol. 31:e70041. doi: 10.1111/gcb.70041, 39840664

[ref40] Lu L. Ding L. Zhang X. Wu B. Chen H. Peng C. . (2025). Shading reduces root aluminum content and restructures epiphytic microbial communities on the subtropical plateau of Southwest China. Pol. J. Environ. Stud. 34, 7675–7688. doi: 10.15244/pjoes/193907

[ref41] Lu J. Li J. Peng P. Chen L. Li Z. Li Y. . (2025). Effects of different varieties of *Camellia oleifera* on soil microbial community structure and stability. J. Soil Sci. Plant Nutr. 25, 4183–4196. doi: 10.1007/s42729-025-02391-2

[ref42] Lv J. Huo C. Zhang J. Huang Y. Su Y. Lv Y. . (2024). Host genotype and age shape the microbial community in the rhizosphere soils of Camellia forests. Front. Microbiol. 15:255. doi: 10.3389/fmicb.2024.1440255, 39411438 PMC11477377

[ref43] Lv X. Jiang W. He F. Yao Y. He J. Chen Y. . (2025). Intercropping changed soil phosphorus fractions and bacterial communities in *Camellia oleifera* plantations. J. Soils Sediments 25, 2360–2370. doi: 10.1007/s11368-025-04088-6

[ref44] Ma X. Liao J. Zhao J. (2023). Experiment and meta-analysis on the effects of grass cultivation in the orchard on fruit yield and quality. Food Sci. Technol. 43:122. doi: 10.1590/fst.95122

[ref45] Malik A. A. Martiny J. B. H. Brodie E. L. Martiny A. C. Treseder K. K. Allison S. D. (2020). Defining trait-based microbial strategies with consequences for soil carbon cycling under climate change. ISME J. 14, 1–9. doi: 10.1038/s41396-019-0510-0, 31554911 PMC6908601

[ref46] Mon W. W. Toma Y. Ueno H. (2024). Combined effects of rice husk biochar and organic manures on soil chemical properties and greenhouse gas emissions from two different paddy soils. Soil Syst. 8:32. doi: 10.3390/soilsystems8010032

[ref47] Muhammad I. Sainju U. M. Zhao F. Khan A. Ghimire R. Fu X. . (2019). Regulation of soil CO_2_ and N_2_O emissions by cover crops: a meta-analysis. Soil Tillage Res. 192, 103–112. doi: 10.1016/j.still.2019.04.020

[ref48] Ning Q. Chen L. Li F. Zhou G. Zhang C. Ma D. . (2023). Tradeoffs of microbial life history strategies drive the turnover of microbial-derived organic carbon in coastal saline soils. Front. Microbiol. 14:1436. doi: 10.3389/fmicb.2023.1141436, 37032859 PMC10076556

[ref49] Pedraza V. Gonzalez-Andujar J. L. (2025). Integrated weed management in olive orchard: the effect of spontaneous grass cover crops on weed community, olive production and soil fertility. Eur. J. Agron. 169:127706. doi: 10.1016/j.eja.2025.127706

[ref50] Qiao H. Chen L. Hu Y. Deng C. Sun Q. Deng S. . (2021). Soil microbial resource limitations and community assembly along a Camellia oleifera plantation Chronosequence. Front. Microbiol. 12:165. doi: 10.3389/fmicb.2021.736165, 34925257 PMC8675945

[ref51] Qiao H. Liu C. Deng C. Sun Q. Deng S. Duan X. . (2024). Microbial metabolic limitation and soil multifunctionality changes across subtropical woodlands in southern China. Forests 15:527. doi: 10.3390/f15030527

[ref52] Ren J. Li F. Yin C. (2023). Orchard grass safeguards sustainable development of fruit industry in China. J. Clean. Prod. 382:135291. doi: 10.1016/j.jclepro.2022.135291

[ref53] Rodriguez-Ramos J. C. Scott N. Marty J. Kaiser D. Hale L. (2022). Cover crops enhance resource availability for soil microorganisms in a pecan orchard. Agric. Ecosyst. Environ. 337:108049. doi: 10.1016/j.agee.2022.108049

[ref54] Shi H. Chen Y. Huang S. Cheng X. Chen H. Xu R. . (2024). Management of hickory forest understory vegetation increases ecosystem carbon sequestration, but it also increases soil greenhouse gas emissions in the short term. Agronomy 14:7675. doi: 10.3390/agronomy14122937

[ref55] Shi C. Wang X. Jiang S. Xu J. Luo J. (2024). Investigating the impact of long-term bristlegrass coverage on rhizosphere microbiota, soil metabolites, and carbon–nitrogen dynamics for pear agronomic traits in orchards. Front. Microbiol. 15:1254. doi: 10.3389/fmicb.2024.1461254, 39301192 PMC11411186

[ref56] Shi X. Wu H. Xie S. Li H. Wang Y. Wang Y. . (2024). Effects of different weeding methods on soil physicochemical properties, root morphology, and fruit economic traits in *Camellia oleifera* Abel. plantations. Horticulturae 10:1093. doi: 10.3390/horticulturae10101093

[ref57] Shirale A. O. Schön J. Gentsch N. Breunig P. (2024). Cover crops support the climate change mitigation potential of agroecosystems. PLoS One 19:139. doi: 10.1371/journal.pone.0302139 PMC11078372 38717995

[ref58] Sun W. Zhao M. Dong T. Ma M. (2025). Effects of different intercropping patterns on soil organic carbon content, enzyme activity, and aggregate stability in apple orchards on the loess plateau. Sci. Rep. 15:34573. doi: 10.1038/s41598-025-18022-w, 41044360 PMC12494687

[ref59] Suo G.-D. Xie Y.-S. Zhang Y. Luo H. (2019). Long-term effects of different surface mulching techniques on soil water and fruit yield in an apple orchard on the loess plateau of China. Sci. Hortic. 246, 643–651. doi: 10.1016/j.scienta.2018.11.028

[ref60] Tang W. Yang H. Wang W. Wang C. Pang Y. Chen D. . (2022). Effects of living grass mulch on soil properties and assessment of soil quality in Chinese apple orchards: a meta-analysis. Agronomy 12:1974. doi: 10.3390/agronomy12081974

[ref61] Tarin M. W. K. Khaliq M. A. Fan L. Xie D. Tayyab M. Chen L. . (2021). Divergent consequences of different biochar amendments on carbon dioxide (CO_2_) and nitrous oxide (N_2_O) emissions from the red soil. Sci. Total Environ. 754:1935. doi: 10.1016/j.scitotenv.2020.141935 32916486

[ref62] Terrer C. Phillips R. P. Hungate B. A. Rosende J. Pett-Ridge J. Craig M. E. . (2021). A trade-off between plant and soil carbon storage under elevated CO_2_. Nature 591, 599–603. doi: 10.1038/s41586-021-03306-8 33762765

[ref63] Tian L. Liu Y. Ma Y. Duan J. Chen F. Deng Y. . (2023). Combined role of ground cover management in altering orchard surface–subsurface erosion and associated carbon–nitrogen-phosphorus loss. Environ. Sci. Pollut. Res. 31, 5655–5667. doi: 10.1007/s11356-023-31535-z, 38123779

[ref64] Tu A. Xie S. Zheng H. Li H. Li Y. Mo M. (2021). Long-term effects of living grass mulching on soil and water conservation and fruit yield of citrus orchard in South China. Agric. Water Manag. 252:106897. doi: 10.1016/j.agwat.2021.106897

[ref65] Ullah S. Wu J. Shah J. A. Wang X. Lyu Y. Guo Z. . (2024). Tree diversity drives understory carbon storage rather than overstory carbon storage across forest types. J. For. Res. 35:125. doi: 10.1007/s11676-024-01776-w

[ref66] Wan P. He R. (2021). Short-term effects of cover grass on soil microbial communities in an apple orchard on the loess plateau. Forests 12:1787. doi: 10.3390/f12121787

[ref67] Wang H. Beule L. Zang H. Pfeiffer B. Ma S. Karlovsky P. . (2020). The potential of ryegrass as cover crop to reduce soil N_2_O emissions and increase the population size of denitrifying bacteria. Eur. J. Soil Sci. 72, 1447–1461. doi: 10.1111/ejss.13047

[ref68] Wang R. Cao B. Sun Q. Song L. (2020). Response of grass interplanting on bacterial and fungal communities in a jujube orchard in Ningxia, Northwest China. Heliyon 6:e03489. doi: 10.1016/j.heliyon.2020.e03489, 32154422 PMC7052399

[ref69] Wang P. Ding L. Li F. Liao J. Wang M. (2022). Herbivore camping reshapes the taxonomy, function and network of pasture soil microbial communities. PeerJ 10:e14314. doi: 10.7717/peerj.14314, 36389419 PMC9653066

[ref70] Wang Y. Huang Q. Gao H. Zhang R. Yang L. Guo Y. . (2021). Long-term cover crops improved soil phosphorus availability in a rain-fed apple orchard. Chemosphere 275:130093. doi: 10.1016/j.chemosphere.2021.130093, 33652274

[ref71] Wang Y. Huang Q. Liu C. Ding Y. Liu L. Tian Y. . (2020). Mulching practices alter soil microbial functional diversity and benefit to soil quality in orchards on the loess plateau. J. Environ. Manag. 271:110985. doi: 10.1016/j.jenvman.2020.110985, 32579532

[ref72] Wang Z. Liu R. Fu L. Tao S. Bao J. (2023). Effects of orchard grass on soil fertility and nutritional status of fruit trees in Korla fragrant pear orchard. Horticulturae 9:903. doi: 10.3390/horticulturae9080903

[ref73] Wang J. Qin X. Tan Y. Du Y. Tudi Y. Yang Y. . (2024). Impact of grass cover on the soil physicochemical properties in China’s orchards: a meta-analysis. Agrofor. Syst. 98, 1745–1758. doi: 10.1007/s10457-024-00985-w

[ref74] Wei H. Zhang K. Zhang J. Li D. Zhang Y. Xiang H. (2018). Grass cultivation alters soil organic carbon fractions in a subtropical orchard of southern China. Soil Tillage Res. 181, 110–116. doi: 10.1016/j.still.2018.04.009

[ref75] Wu Y. Wang X. Hu R. Zhao J. Jiang Y. (2021). Responses of soil microbial traits to ground cover in Citrus orchards in Central China. Microorganisms 9:122507. doi: 10.3390/microorganisms9122507, 34946109 PMC8708208

[ref76] Xiang Y. Chang S. X. Shen Y. Chen G. Liu Y. Yao B. . (2023). Grass cover increases soil microbial abundance and diversity and extracellular enzyme activities in orchards: a synthesis across China. Appl. Soil Ecol. 182:104720. doi: 10.1016/j.apsoil.2022.104720

[ref77] Xiang Y. Li Y. Liu Y. Zhang S. Yue X. Yao B. . (2022). Factors shaping soil organic carbon stocks in grass covered orchards across China: a meta-analysis. Sci. Total Environ. 807:632. doi: 10.1016/j.scitotenv.2021.150632, 34606865

[ref78] Xiang Q. Ma T. Wang X. Yang Q. Lv L. Wang R. . (2024). Effects of different living grass mulching on soil carbon and nitrogen in an apple orchard on loess plateau. Agronomy 14:1917. doi: 10.3390/agronomy14091917

[ref79] Xiao L. Lai S. Chen M. Long X. Fu X. Yang H. (2022). Effects of grass cultivation on soil arbuscular mycorrhizal fungi community in a tangerine orchard. Rhizosphere 24:583. doi: 10.1016/j.rhisph.2022.100583

[ref80] Xu H. Huang X. Chen J. Chen Y. Wang Y. Wu X. . (2023). Intercropping with legumes alleviates soil N limitation but aggravates P limitation in a degraded agroecosystem as shown by ecoenzymatic stoichiometry. Soil Biol. Biochem. 187:109210. doi: 10.1016/j.soilbio.2023.109210

[ref81] Xue X. Chen R. Xu C. Zhang C. Dong L. Zhao X. . (2023). Apple-marigold intercropping improves soil properties by changing soil metabolomics and bacterial community structures. Front. Microbiol. 14:1195985. doi: 10.3389/fmicb.2023.1195985, 37455738 PMC10343436

[ref82] Yao S. Merwin I. A. Bird G. W. Abawi G. S. Thies J. E. (2005). Orchard floor management practices that maintain vegetative or biomass groundcover stimulate soil microbial activity and alter soil microbial community composition. Plant Soil 271, 377–389. doi: 10.1007/s11104-004-3610-0

[ref83] Ye H.-L. Chen Z.-G. Jia T.-T. Su Q.-W. Su S.-C. (2021). Response of different organic mulch treatments on yield and quality of *Camellia oleifera*. Agric. Water Manag. 245:106654. doi: 10.1016/j.agwat.2020.106654

[ref84] Ye H. Wen Y. Chen Z. Zhang T. Li S. Guan M. . (2022). Relationship of soil microbiota to seed kernel metabolism in Camellia oleifera under mulched. Front. Plant Sci. 13:604. doi: 10.3389/fpls.2022.920604, 35795350 PMC9251579

[ref85] Zang X. Li K. Yun T. Rashed A. A. Melebari D. M. Ding Z. . (2025). Comparison between tropical legumes and natural grasses in improving tropical rainforest soil health: a case study in guava (*Psidium Guajava* L.) orchards. BMC Plant Biol. 25:6395. doi: 10.1186/s12870-025-06395-z, 40133796 PMC11934814

[ref86] Żelazny W. R. Licznar-Małańczuk M. (2018). Soil quality and tree status in a twelve-year-old apple orchard under three mulch-based floor management systems. Soil Tillage Res. 180, 250–258. doi: 10.1016/j.still.2018.03.010

[ref87] Zhang A. Cheng G. Hussain Q. Zhang M. Feng H. Dyck M. . (2017). Contrasting effects of straw and straw–derived biochar application on net global warming potential in the loess plateau of China. Field Crop Res. 205, 45–54. doi: 10.1016/j.fcr.2017.02.006

[ref88] Zhao Y. Gao C. Rong Z. Zhao C. (2021). Understory vegetation should not be ignored in the estimation of forest carbon stocks in Qilian Mountains National Nature Reserve. Acta Ecol. Sin. 41, 318–324. doi: 10.1016/j.chnaes.2020.06.009

[ref89] Zhao C. Gao B. Wang L. Huang W. Xu S. Cui S. (2021). Spatial patterns of net greenhouse gas balance and intensity in Chinese orchard system. Sci. Total Environ. 779:146250. doi: 10.1016/j.scitotenv.2021.146250, 33744568

[ref90] Zhao H. Lakshmanan P. Wang X. Xiong H. Yang L. Liu B. . (2022). Global reactive nitrogen loss in orchard systems: a review. Sci. Total Environ. 821:153462. doi: 10.1016/j.scitotenv.2022.153462, 35093357

[ref91] Zheng W. Gong Q. Zhao Z. Liu J. Zhai B. Wang Z. . (2018). Changes in the soil bacterial community structure and enzyme activities after intercrop mulch with cover crop for eight years in an orchard. Eur. J. Soil Biol. 86, 34–41. doi: 10.1016/j.ejsobi.2018.01.009

[ref92] Zheng W. Hu L. Peng Y. Wu J. Yan W. (2024). Effect of peanut straw mulching on the soil nitrogen change and functional genes in the *Camellia oleifera* intercropping system. J. Soils Sediments 24, 3473–3484. doi: 10.1007/s11368-024-03896-6

[ref93] Zheng J. Y. Zhao J. S. Shi Z. H. Wang L. (2021). Soil aggregates are key factors that regulate erosion-related carbon loss in citrus orchards of southern China: bare land vs. grass-covered land. Agric. Ecosyst. Environ. 309:107254. doi: 10.1016/j.agee.2020.107254

[ref94] Zhou J. Ning D. (2017). Stochastic community assembly: does it matter in microbial ecology? Microbiol. Mol. Biol. Rev. 81:e00002-e00017. doi: 10.1128/mmbr.00002-17, 29021219 PMC5706748

[ref95] Zhuo Z. Li H. Zhou B. Wang X. Lin X. Wang L. . (2025). Effects of grass cultivation on the soil organic carbon of citrus orchard soil and their microbial mechanisms in red soil hilly areas. J. Soil Sci. Plant Nutr. 25, 4880–4896. doi: 10.1007/s42729-025-02435-7

