# Supplementary material for: Legume cover under Camellia oleifera forests enhances understory biomass carbon storage and soil CO2 flux but declines soil inorganic carbon storage on a karst steep slope
Source: Front Microbiol. 2026 Jan 26;16:1714945. doi: 10.3389/fmicb.2025.1714945 (PMC12883653; doi:10.3389/fmicb.2025.1714945)
Supplement: Supplementary file 1 [file Data_Sheet_1.pdf]

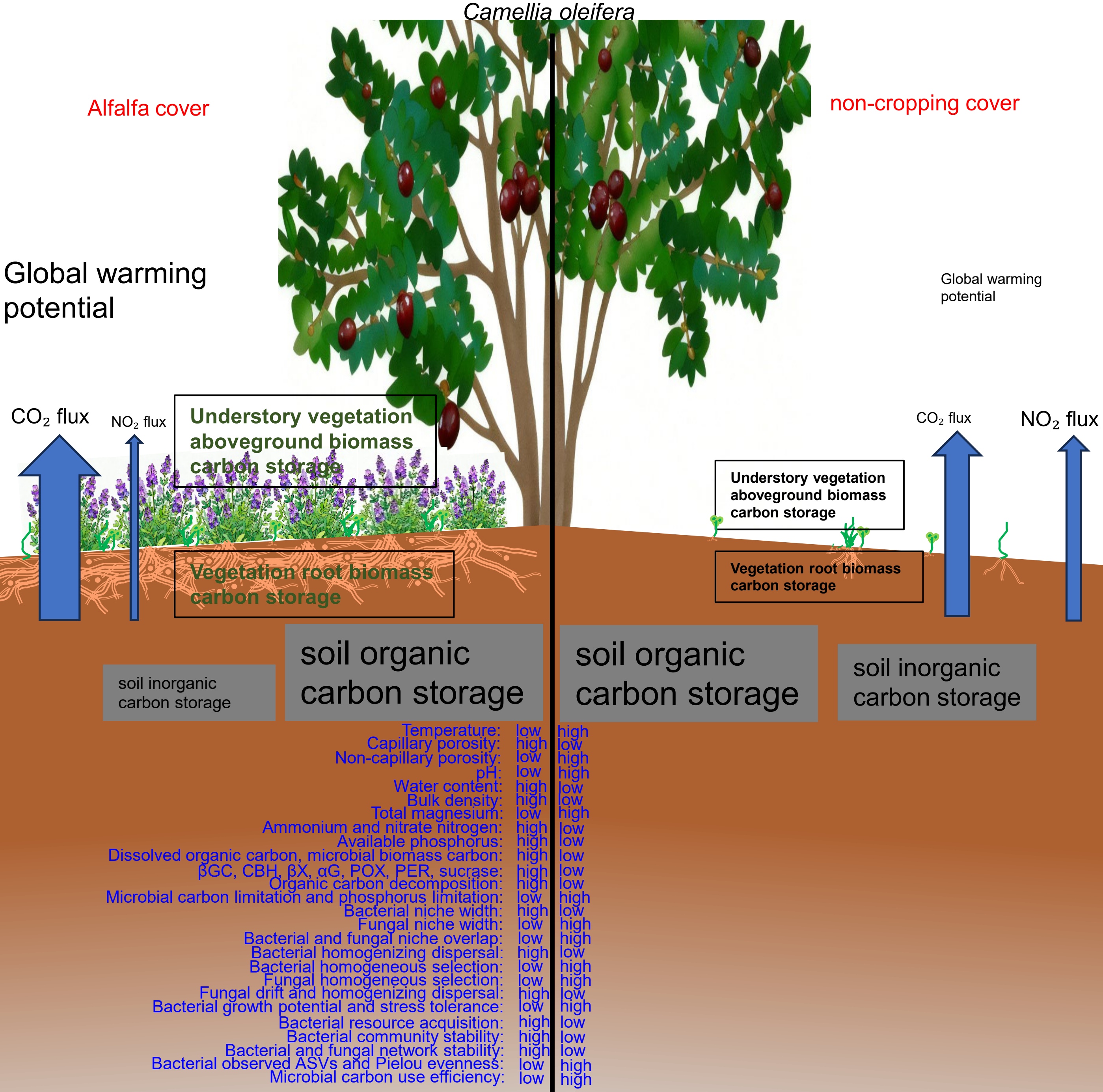

Camellia oleifera
Alfalfa cover
non-cropping cover
Global warming potential
Global warming potential
CO_2 flux
NO_2 flux
Understory vegetation aboveground biomass carbon storage
Vegetation root biomass carbon storage
CO_2 flux
NO_2 flux
Understory vegetation aboveground biomass carbon storage
Vegetation root biomass carbon storage
soil inorganic carbon storage
soil organic carbon storage
soil organic carbon storage
soil inorganic carbon storage
Temperature: low high
Capillary porosity: high low
Non-capillary porosity: low high
pH: low high
Water content: high low
Bulk density: high low
Total magnesium: low high
Ammonium and nitrate nitrogen: high low
Available phosphorus: high low
Dissolved organic carbon, microbial biomass carbon: high low
βGC, CBH, βX, αG, POX, PER, sucrase: high low
Organic carbon decomposition: high low
Microbial carbon limitation and phosphorus limitation: low high
Bacterial niche width: high low
Fungal niche width: low high
Bacterial and fungal niche overlap: low high
Bacterial homogenizing dispersal: high low
Bacterial homogeneous selection: low high
Fungal homogeneous selection: low high
Fungal drift and homogenizing dispersal: high low
Bacterial growth potential and stress tolerance: low high
Bacterial resource acquisition: high low
Bacterial community stability: high low
Bacterial and fungal network stability: high low
Bacterial observed ASVs and Pielou evenness: low high
Microbial carbon use efficiency: low high
