# Supplementary figures and images for: Legume cover under Camellia oleifera forests enhances understory biomass carbon storage and soil CO2 flux but declines soil inorganic carbon storage on a karst steep slope
Source: Front Microbiol. 2026 Jan 26;16:1714945. doi: 10.3389/fmicb.2025.1714945 (PMC12883653; doi:10.3389/fmicb.2025.1714945)

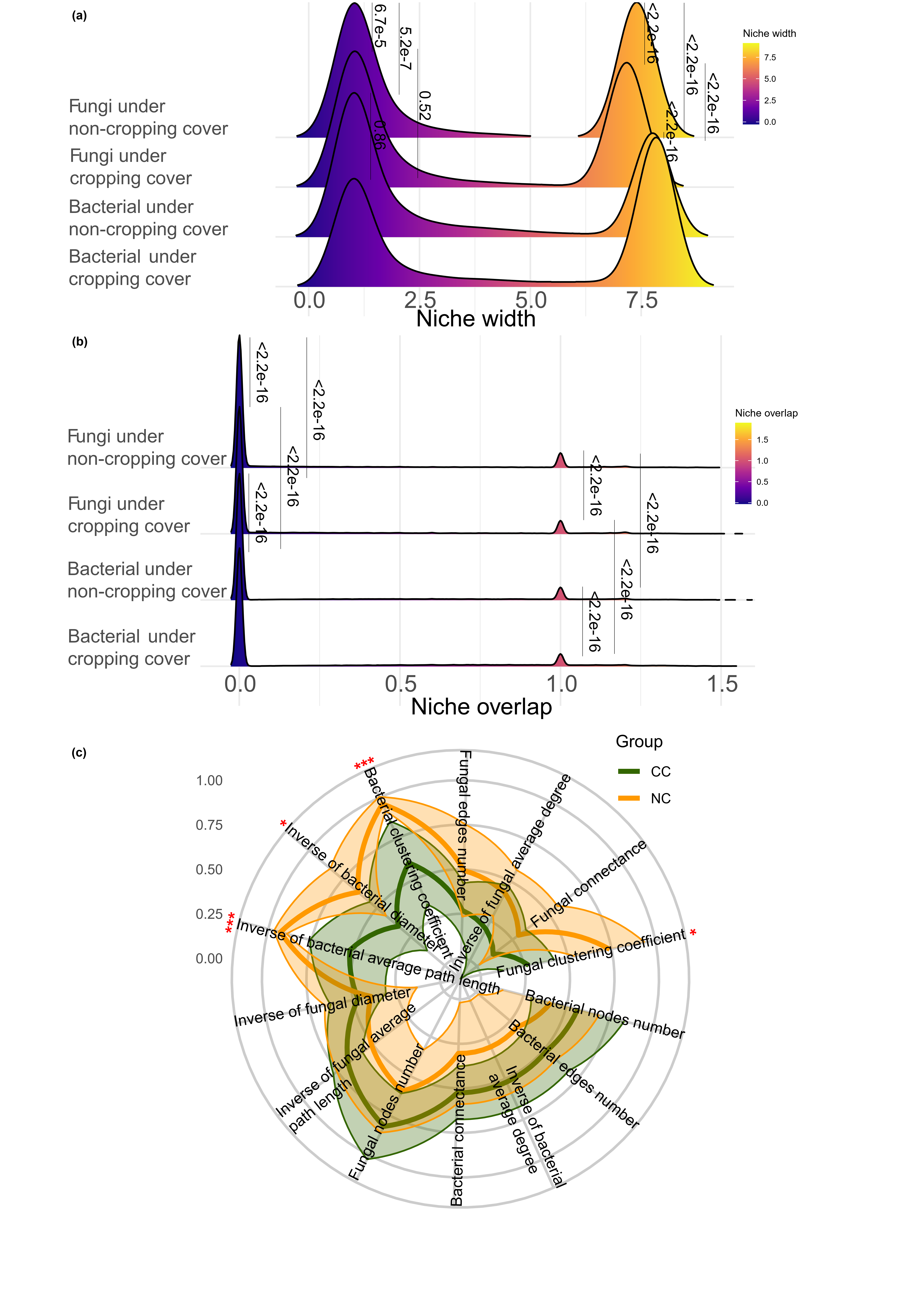

Supplement: Supplementary file 2 [file Image_1.tif]

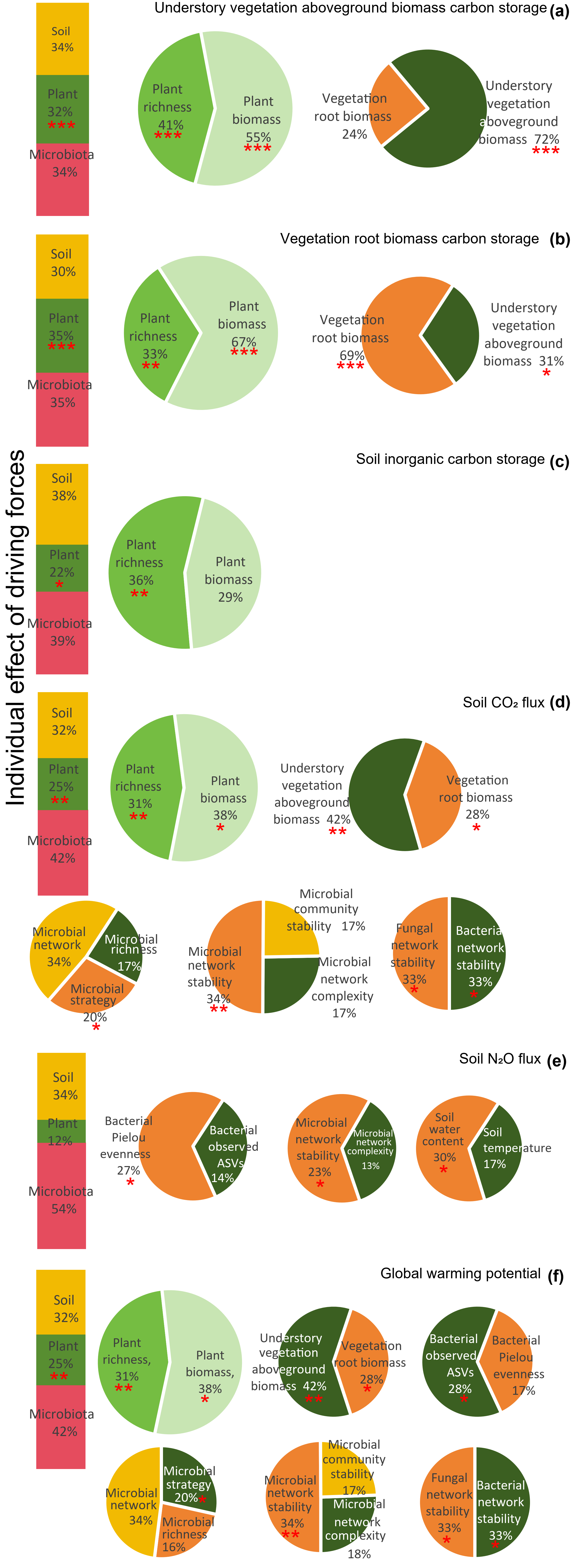

Supplement: Supplementary file 3 [file Image_2.tif]
